# Essential role of GEXP15, a specific Protein Phosphatase type 1 partner, in *Plasmodium berghei* in asexual erythrocytic proliferation and transmission

**DOI:** 10.1371/journal.ppat.1007973

**Published:** 2019-07-26

**Authors:** Thomas Hollin, Caroline De Witte, Aline Fréville, Ida Chiara Guerrera, Cerina Chhuon, Jean-Michel Saliou, Fabien Herbert, Christine Pierrot, Jamal Khalife

**Affiliations:** 1 Univ. Lille, CNRS, Inserm, CHU Lille, Institut Pasteur de Lille, U1019 - UMR8204 – CIIL - Center for Infection and Immunity of Lille, Lille, France; 2 Proteomics platform 3P5-Necker, Université Paris Descartes - Structure Fédérative de Recherche Necker, INSERM US24/CNRS UMS3633, Paris, France; Francis Crick Institute, UNITED KINGDOM

## Abstract

The essential and distinct functions of Protein Phosphatase type 1 (PP1) catalytic subunit in eukaryotes are exclusively achieved through its interaction with a myriad of regulatory partners. In this work, we report the molecular and functional characterization of Gametocyte EXported Protein 15 (GEXP15), a *Plasmodium* specific protein, as a regulator of PP1. *In vitro* interaction studies demonstrated that GEXP15 physically interacts with PP1 through the RVxF binding motif in *P*. *berghei*. Functional assays showed that GEXP15 was able to increase PP1 activity and the mutation of the RVxF motif completely abolished this regulation. Immunoprecipitation assays of tagged GEXP15 or PP1 in *P*. *berghei* followed by immunoblot or mass spectrometry analyses confirmed their interaction and showed that they are present both in schizont and gametocyte stages in shared protein complexes involved in the spliceosome and proteasome pathways and known to play essential role in parasite development. Phenotypic analysis of viable GEXP15 deficient *P*. *berghei* blood parasites showed that they were unable to develop lethal infection in BALB/c mice or to establish experimental cerebral malaria in C57BL/6 mice. Further, although deficient parasites produced gametocytes they did not produce any oocysts/sporozoites indicating a high fitness cost in the mosquito. Global proteomic and phosphoproteomic analyses of GEXP15 deficient schizonts revealed a profound defect with a significant decrease in the abundance and an impact on phosphorylation status of proteins involved in regulation of gene expression or invasion. Moreover, depletion of GEXP15 seemed to impact mainly the abundance of some specific proteins of female gametocytes. Our study provides the first insight into the contribution of a PP1 regulator to *Plasmodium* virulence and suggests that GEXP15 affects both the asexual and sexual life cycle.

## Author summary

In the absence of an effective vaccine and the emerging resistance to artemisinin combination therapy, malaria is still a significant threat to human health. Increasing our understanding of the specific mechanisms of the biology of *Plasmodium* is essential to propose new strategies to control this infection. Here, we demonstrated that GEXP15, a specific protein in *Plasmodium*, was able to interact with the Protein Phosphatase 1 and regulate its activity. We showed that both proteins are implicated in common protein complexes involved in the mRNA splicing and proteasome pathways. We reported that the deletion of GEXP15 leads to a loss of parasite virulence during asexual stages and a total abolishment of the capacity of deficient parasites to develop in the mosquito. We also found that this deletion affects both protein phosphorylation status and significantly decreases the expression of essential proteins in schizont and gametocyte stages. This study characterizes for the first time a novel molecular pathway through the control of PP1 by an essential and specific *Plasmodium* regulator, which may contribute to the discovery of new therapeutic targets to control malaria.

## Introduction

Convergent findings from several studies indicate that protein dephosphorylation by phosphatases governs key fundamental processes in the biology of eukaryotic cells including *Plasmodium falciparum* [1]. Among these phosphatases, the Protein Phosphatase type 1 catalytic subunit (PP1c) seems to play a pivotal role in the development and growth of *Plasmodium* blood stage parasites [2,3]. In addition, it has been reported in eukaryotes that several conserved PP1c interacting proteins (PIPs) endowed with phosphatase regulatory functions are as essential as PP1c itself [4–7]. In mammalian cells, hundreds of PIPs have been identified and classified as regulators and substrates, capable of targeting PP1c to particular cellular organelles [8,9]. The combination of PP1c with an extensive range of PIPs contributes to the constitution of specific PP1 network, playing an effective role as a hub in diverse cellular functions.

Our earlier studies on *P*. *falciparum* revealed the expression of four conserved putative regulators (PfLRR1, Pf Inhibitor 2, Pf Inhibitor 3 and Pfeif2β) which have been extensively explored to define their functions [10–15]. In this context, biochemical and structure-activity relationship studies demonstrated that these regulators bind to recombinant PfPP1 and three of them regulate its activity *in vitro*. Several binding sites have also been defined by structure-interaction studies using mutated recombinant proteins and derived peptides, showing the involvement of LRR/LRR cap motifs for PfLRR1 and the consensus RVxF motif for PfI2, PfI3 and Pfeif2β. In addition, knock-out approaches suggested their essentiality in the life cycle of blood stage parasites. Finally, these interactions seem to be indispensable since disrupting the interaction between PP1c and PfI2, PfI3 or PfLRR1 by the use of interfering peptides that inhibit complex formation led to parasite growth inhibition *in vitro* [12–14]. This proof of concept offers a robust basis to consider PP1c interactions as valuable therapeutic targets.

Based on the above data and on the atypical life cycle of *Plasmodium* with ~60% of unknown genes in its genome [16], including specific proteins submitted to reversible phosphorylation, our working hypothesis was that *Plasmodium* must express specific partners, regulators and/or transporters which direct PP1 activity. In order to identify PfPP1 interactors, extensive screens for PfPP1 binding proteins were carried out, using the yeast two-hybrid (Y2H) system combined with direct interactions studies using recombinant proteins [17]. A total of 31 proteins in the correct translational reading frame with the Gal4 activating domain were identified. Upon close inspection of the amino acid sequences of binding regions of these proteins, six clones were identified exhibiting a potential RVxF binding motif with a more restrictive and specific consensus sequence of PP1 interactors [17,18]. Indeed, we have used the PfPP1 interactome to reevaluate the features of flanking residues of the RVxF motif. We observed enrichment for basic amino acids (R and K) at the N-terminal side of this binding motif.

We prioritized candidate genes that were *Plasmodium*-specific with a robust interaction with PfPP1. One of these genes, formerly described as *P*. *falciparum* Gametocyte EXported Protein 15 (PfGEXP15) (PF3D7_1031600), was repeatedly detectable under stringent conditions of binding to PfPP1 throughout the Y2H screens. In addition, the presence of a potential restrictive binding motif was identified in the interacting region of PfGEXP15 [17]. PfGEXP15 has been classified in the GEXP cluster, grouping different proteins predicted to be exported and detected only in the proteome of early gametocytes or being overexpressed at this stage [19]. However, interestingly, PfGEXP15 was also detected in the proteomes of asexual stages and sporozoites suggesting its involvement at different steps of the parasite life cycle [20].

In this work, we confirm that GEXP15 interacts with PP1 and showed its capacity to regulate the dephosphorylation activity of PP1 via a major contribution of RVxF binding motif in *P*. *berghei*. Interatomic analysis revealed that the GEXP15-PP1 are part of two protein complexes involved in mRNA splicing and proteasome pathways, known to play key roles in parasite development. Further, for the first time, we demonstrate that the complete disruption of GEXP15 generates attenuated parasites both for asexual and sexual growth in mice and mosquitoes respectively, suggesting specific and essential functions. Finally, we provide evidence that proteome and phosphoproteome of deficient schizont and gametocyte parasite stages are impacted at different levels arguing for an involvement of GEXP15 in these processes.

## Results

### Interaction of *Plasmodium* GEXP15 with PP1 and binding motifs

In order to extend and to further characterize the GEXP15 that we previously reported as a partner of PP1 in *P*. *falciparum* [17], its ortholog in *P*. *berghei* (PBANKA_0515400) was investigated. The predicted PbGEXP15 protein (656 aa) shares an overall identity of 41% with PfGEXP15 and both contain two putative consensus RVxF binding motifs to PP1 (S1 Fig). The construct of PbGEXP15 _4–590_, spanning almost the full-length sequence was tested in the Y2H system in which PfPP1c was used as bait as it exhibits 99% identity with PbPP1. We observed that only the diploids expressing PbGEXP15 and PfPP1c were able to grow on selective and stringent media indicating their ability to specifically interact with each other (S2A Fig). Control constructs (empty vector, vector expressing unrelated gene) did not show any growth. To better define the interacting regions, constructs with PbGEXP15 _4–178_ containing the first RVxF binding motif to PP1 or PbGEXP15 _446–596_ comprising the second one were used. PbGEXP15 _4–178_ revealed the same specific interaction with PfPP1c, confirming the data described above (S2B Fig). In the case of PbGEXP15 _446–596_ with the second RVxF motif no yeast growth was observed, suggesting that it is a random sequence and a non-canonical binding site (S2B Fig). To validate the involvement of the first RVxF binding motif present in PbGEXP15, site directed mutagenesis was carried out to obtain V34A and F36A (PbGEXP15 _4–178 KKKKKAQA_). Interaction with PfPP1c was not observed with this mutant, confirming that the native sequence represents a genuine RVxF binding motif (S2B Fig). The main contribution of this RVxF motif was reinforced by the lack of growth on high stringency selection in the presence of PfPP1c mutated at its docking site by replacing F255 and F256 by two alanine residues (S2C Fig) [18,21–23].

**Fig 1:**
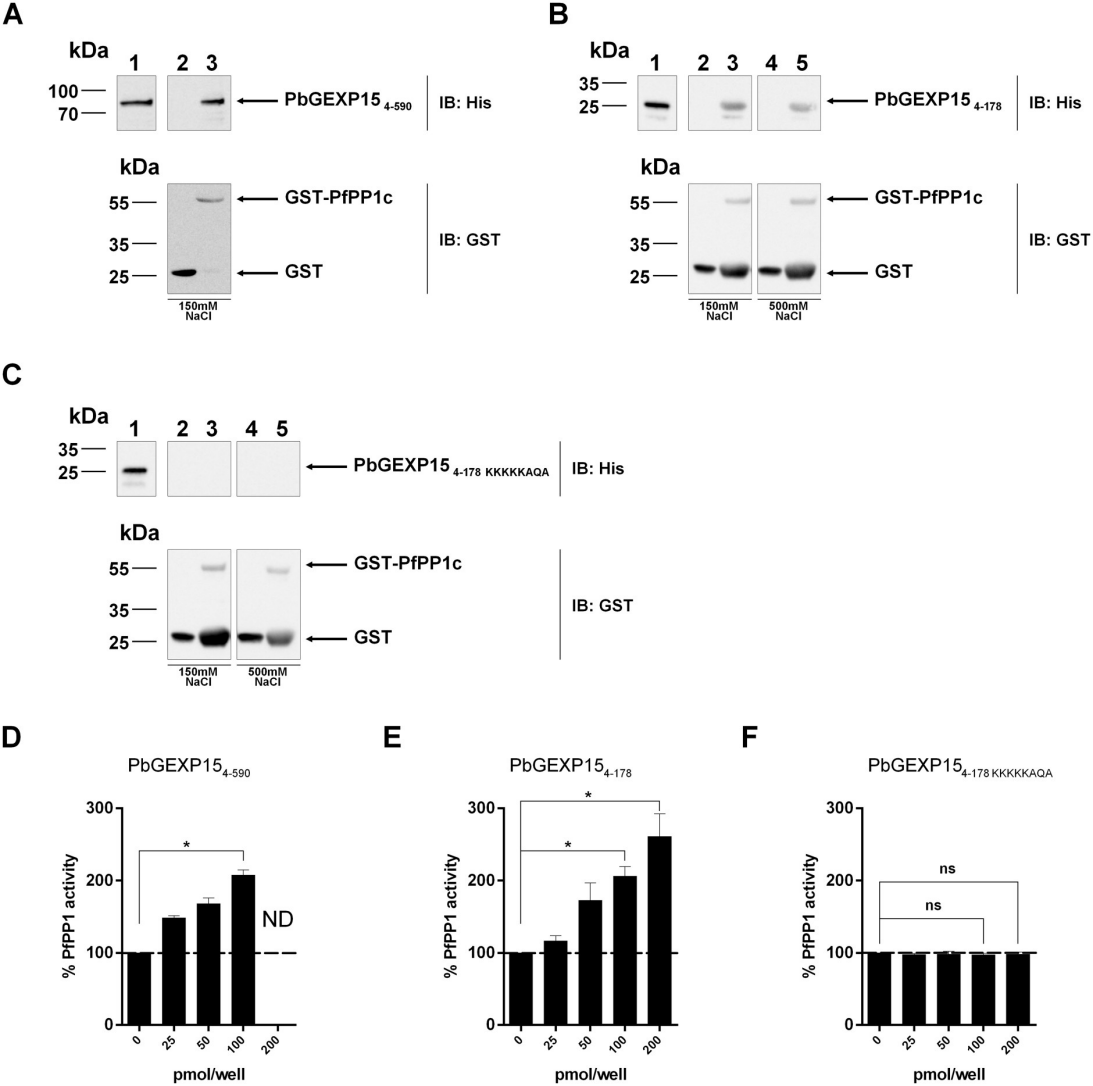
Direct interaction of PbGEXP15 with PP1c and its effect on the phosphatase activity *in vitro*. A, B and C represent GST-pull down experiments. A, Immunoblot (IB) represents the input positive control (500ng) in lane 1 and the GST-pull down of 6-His PbGEXP15 _4–590_ with GST alone or PfPP1c-GST in lanes 2 and 3 respectively and revealed with mAb anti-His (upper panel) and anti-GST (lower panel). B, Immunoblot (IB) represents the input positive control (500ng) in lane 1 and the GST-pull down of 6-His PbGEXP15 _4–178_ with GST alone or PfPP1c-GST in lanes 2 and 3 respectively in the presence of 150 mM NaCl and revealed with mAb anti-His (upper panel) and anti-GST (lower panel). Lanes 4 and 5 represent the GST-pull down as in lanes 2 and 3 in the presence of 500 mM NaCl. C, 6-His PbGEXP15 _4–178 KKKKKAQA_ was incubated in the same conditions as in B. Figures D, E and F show the impact on phosphatase activity. PfPP1c was pre-incubated for 30 min at 37°C with different concentrations of PbGEXP15 _4–590_ (D), PbGEXP15 _4–178_ (E) and PbGEXP15 _4–178 KKKKKAQA_ (F) before the addition of pNPP. The optical density was measured after 1h at 37°C. Results indicate the mean ± SD of the % of relative increase from two independent experiments performed in duplicate. Mann–Whitney *U* test was performed for 100 and 200 pmol/well of recombinant proteins compared to control, * p<0.05. ND: Not determined. ns: no significant.

To further support the PbGEXP15-PP1 interaction, GST-PfPP1c pull-down experiments with wild-type and mutated His-PbGEXP15 recombinant proteins were carried out. Western blot analyses showed that PbGEXP15 _4–590_ (Fig 1A), and PbGEXP15 _4–178_ (Fig 1B) bound to GST-PfPP1c but not to GST alone, while the mutated PbGEXP15 _4–178 KKKKKAQA_ revealed no binding to GST-PfPP1c (Fig 1C). In addition, the interaction of PbGEXP15 _4–178_ with PfPP1c was tested under stringent conditions (500 mM NaCl) and remained detectable. These data showed that GEXP15 physically and strongly binds to PP1c via the RVxF motif.

### Effect of GEXP15 on the activity of PP1

Based on the ability of recombinant GEXP15 to interact directly with PP1c, its effect on the phosphatase activity was assessed. As depicted in Fig 1D and 1E, using a quantity of PfPP1c generating a linear release of phosphate from pNPP substrate, the addition of either PbGEXP15 _4–590_ or a shorter PbGEXP15 _4–178_ protein strongly increased the dephosphorylation activity of PfPP1c in a concentration-dependent manner. A two-fold increase was observed at 100 pmol/well with both versions of PbGEXP15 proteins, suggesting that the main activating region of PfPP1c is carried by the N-terminal moiety of GEXP15. The use of the PbGEXP15 _4–178 KKKKKAQA_ mutant abolished the regulatory effect on PP1 activity (Fig 1F). These data exclude any non-specific activation of PfPP1c and support the major role of RVxF in the function of GEXP15.

### Localization of PbGEXP15 and PbPP1 and detection of the complex in *P*. *berghei*

**Fig 2:**
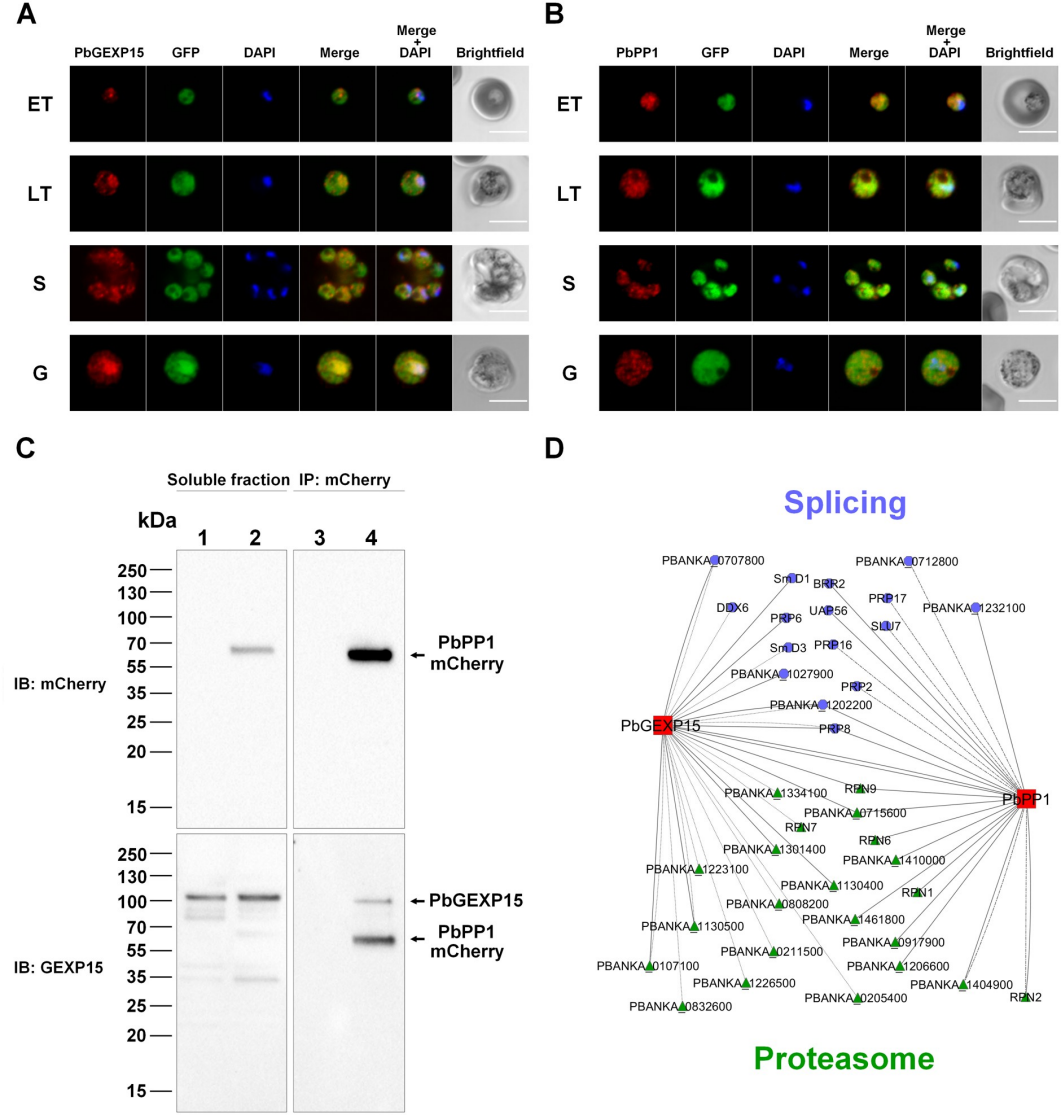
Localization and interactomes of PbGEXP15 and PbPP1 in *P*. *berghei*. Immunofluorescence assay of PbGEXP15-mCherry (A) and PbPP1-mCherry (B) on early trophozoite (ET), late trophozoite (LT), schizont (S) and gametocyte (G) stages. The PbGEXP15 or PbPP1 were labeled with anti-mCherry (red) and were detected within the parasite (GFP signal). Parasite nuclei are stained with DAPI (blue). Merged images represents the composite of PbGEXP15-mCherry or PbPP1-mCherry along with GFP. Scale bar: 5 μm. (C) Detection of endogenous PbGEXP15 and PbPP1-mCherry from parental (lanes 1 and 3) and transfected *P*. *berghei* parasites (lanes 2 and 4). The different soluble protein extracts (lanes 1 and 2) were used with mCherry-beads to immunoprecipitate (IP) PbPP1-mCherry (lanes 3 and 4). Immunoblot (IB) was performed using anti-mCherry (upper panel) then stripped and probed with anti-GEXP15 (lower panel). Note that the signal corresponding to PbPP1-mCherry is still detectable after stripping and reprobing with anti-GEXP15 antisera. (D) Immunoprecipitations followed by mass spectrometry analysis (IP/MS) identified two common pathways between PbGEXP15 and PbPP1 (in red). The RNA splicing network is represented in blue octagons and the proteasome in green triangles. Interactions from IP/MS in PbGEXP15-mCherry and PbPP1-HA schizonts are indicated in solid lines and those coming from PbGEXP15-mCherry gametocytes in dotted lines. To complete the network, the *P*. *berghei* orthologs to the partners of PP1 previously identified in *P*. *falciparum* are included and represented in dash-dotted lines [17]. The image was generated with Cytoscape 3.

To follow up the localization of PbGEXP15 in blood stages, we generated in GFP-*P*. *berghei* lines [24], parasites expressing PbGEXP15-mCherry or PbPP1-mCherry (S3A and S3B Fig). The expression of these tagged proteins was checked by immunoblots using anti-mCherry antibody (S3C and S3D Fig). Examination of PbGEXP15-mCherry by immunofluorescence assays showed a distribution in the cytoplasm of all parasite stages examined along with clear punctate localization, suggesting potential cytoplasmic organelle structures (Fig 2A). Further, the PbGEXP15 signal clearly exhibited a pattern adjacent to and in the nucleus of trophozoite and gametocyte stages. With respect to PbPP1, the signal was observed throughout the cytoplasm with fluorescence partially overlapping DNA (Fig 2B). Earlier works reported similar distributions for GEXP15 and PP1 in *P*. *falciparum* [10,25], suggesting that the two proteins localize to the same compartments and could interact *in vivo* with each other. To confirm this, an anti-PbGEXP15 antibody raised against the recombinant protein and recognizing the native protein (~100kDa) (S3E Fig) was tested on eluates immunoprecipitated from PbPP1-mCherry parasite extracts with anti-mCherry antibody. As shown in Fig 2C, immunoblot analysis revealed the presence of PbGEXP15. These data, together with the results reported above, strongly support a physical interaction within the parasite.

### Identification of PbGEXP15 interacting proteins and common pathways with PbPP1

In order to provide new information about the functional pathways involving GEXP15, it was important to better define the supramolecular architecture of PbGEXP15 complexes. To this end and to characterize a potential dynamic interactome of GEXP15, we performed global immunoprecipitation of PbGEXP15-mCherry obtained from schizont and gametocyte soluble extracts using anti-mCherry antibody followed by mass spectrometry analysis (IP/MS). With respect to the schizont stages, bait recovery from the IP/MS of 3 biological replicates yielded between 72 and 232 spectral counts with an average of 47% coverage, supporting the selectivity of this approach (S1 Table). Results were validated and filtered if proteins were detected in at least two biological replicates out of three with ≥ 2 peptides and with peptides and spectra ≥ 2 fold compared with the control parental strain. In total, 18 proteins were identified including PbPP1 (S1 Table). This supports the western blot analysis, confirms the endogenous interaction between the two proteins and demonstrates the reliability of the IP/MS approach. According to their GO annotations, the majority of the partners (12/18) are linked to mRNA splicing (6 proteins) or proteasome core complex (6 proteins) indicating at least two networks around PbGEXP15. In the case of the IP/MS in gametocytes, results from two biological replicates have been filtered as described above and we identified 37 proteins (S2 Table). We noticed an overlap of 6 partners already detected in the schizont stage and being mainly involved in splicing and the proteasome. In addition to these proteins, 8 novel members of the proteasome core were identified as well as DDX6 and SmD3 in the mRNA splicing complex confirming the link of PbGEXP15 with these pathways. Regarding PbPP1, it was clearly detected in one replicate and at the limit of cut-off criteria in the second replicate (S2 Table, sheet 2).

To further identify potential shared pathways in the PP1-GEXP15 complexes, we took advantage of a *P*. *berghei* strain expressing PbPP1-HA and completed the PP1 interactome in schizont stages obtained by IP/MS [23]. In this initial work, we confirmed that PbPP1-HA binds with the two most conserved regulators LRR1 and inhibitor 2, interactions previously reported in *P*. *falciparum* [10,12], and a novel regulator designated as RCC-PIP [23]. In this study, further analysis of the PbPP1 interactome was performed. As could be expected given the high number of biological processes implicating PP1, a total of 178 proteins, including PbGEXP15, were identified in this IP/MS based on the cut-off criteria used above (S3 Table). These data revealed that the most important network corresponds to the 60S and 40S ribosomal proteins with the detection of 21 and 18 partners respectively and is consistent with outcomes obtained in different organisms [26–31]. When these data were compared with those obtained with the PfPP1 interactome [17], we observed 18 overlapping proteins. However, other partners can be added since they share similar functions such as HSP/chaperones, 60/40S ribosomal proteins, histones and transcription factors.

Most interestingly, comparative analysis of GEXP15 and PP1 interacting proteins revealed that both proteins are part of common protein complexes (Fig 2D). Indeed, 5 and 10 proteins identified in the PbPP1 interactome are known to be part of the mRNA splicing and the proteasome complexes respectively, suggesting that the GEXP15/PP1 complex is a component of two different networks. Of note, previous studies have highlighted the importance of PP1 in these processes in various organisms, including *P*. *falciparum* [17,26,32–35].

### Disruption of the GEXP15 gene

To investigate the function of GEXP15, a complete disruption of *gexp15* in *P*. *berghei* by double homologous recombination was attempted. A construct comprising 5’ and 3’ UTRs of *pbgexp15* flanking the pyrimethamine-resistance cassette was used for selection after parasite transfection (S4A Fig). Among the pyrimethamine-resistant blood parasites, two clones were selected (Δgexp15cl1 and Δgexp15cl2) and the presence of the double crossover was confirmed along with the absence of the wild locus (S4A Fig). We also performed immunoblot experiments using anti-GEXP15 antisera on schizont parasites. PbGEXP15 protein was only detected in parental, but not in the Δgexp15 parasites, demonstrating a lack of PbGEXP15 protein expression in these clones (S4B Fig).

**Fig 3:**
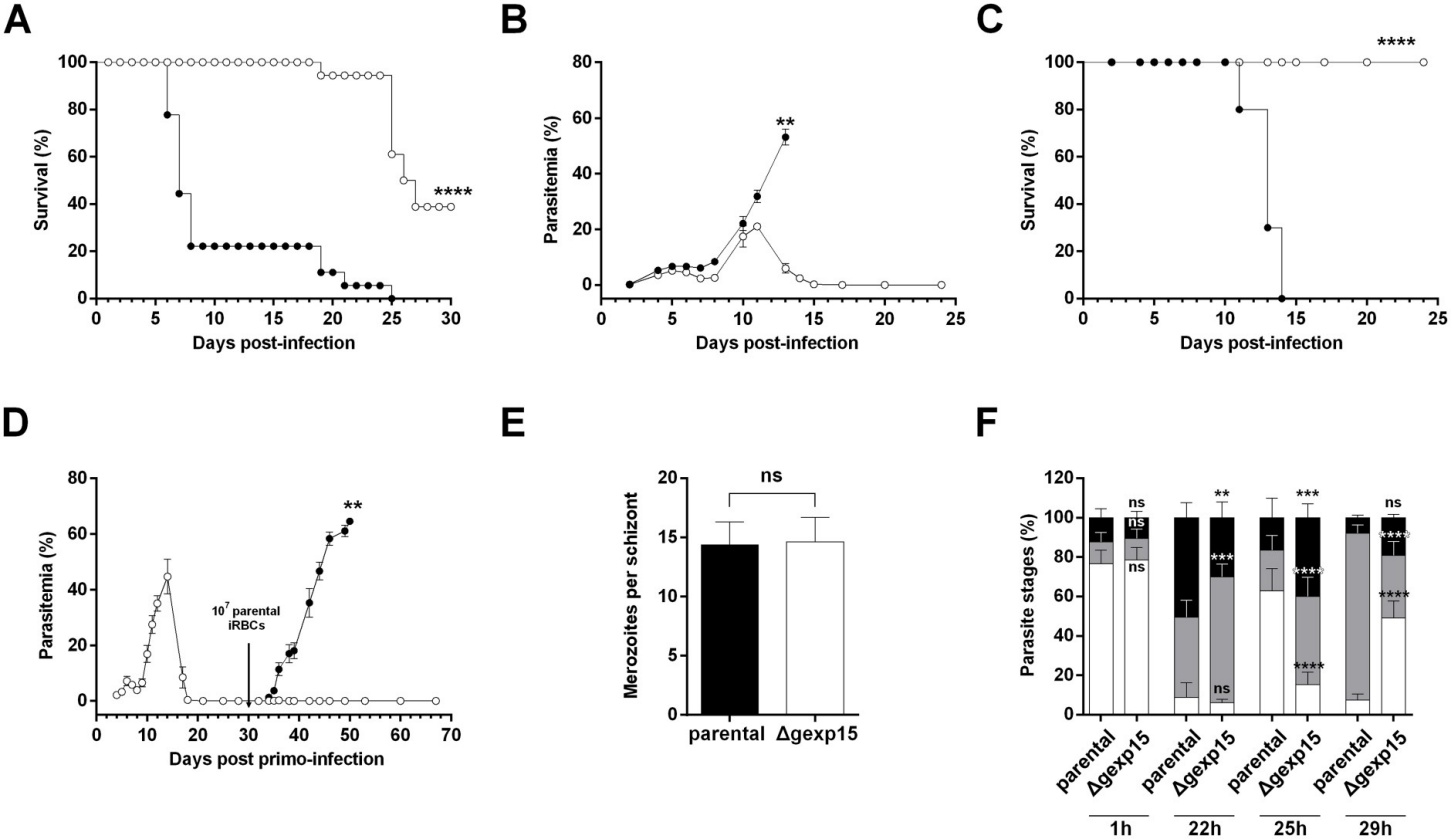
Effect of PbGEXP15 knock-out on parasite virulence and fitness. (A) C57BL/6 mice were intraperitoneally inoculated with 10^6^ of parental (black circles) or Δgexp15 (white circles) infected red blood cells (iRBCs). The cumulative survival rates of three independent experiments (n = 6/experiment, see Table 1) are indicated (Log-rank test, **** p<0.0001). (B) BALB/c mice were inoculated in the same conditions and the course of infection was observed by blood smears. The results of one representative experiment out of two are shown as the mean parasitemia ± SEM (n = 5/experiment, see Table 1) (Wilcoxon test, ** p<0.01). (C) Cumulative survival rates of BALB/c mice were indicated in a second graph (Log-rank test, **** p<0.0001). (D) BALB/c mice were inoculated with 10^6^ of Δgexp15 iRBCs at day 0 (white circles). After parasite clearance, the mice (white circles) and matched naïve mice of the same age (black circles) were inoculated with 10^7^ of parental iRBCs at day 30 (black arrow). The results of one out of two representative experiments are shown as the mean parasitemia ± SEM (n = 11 in total, see Table 1) (Wilcoxon test, ** p<0.01). (E) Number of merozoites per schizont obtained after *in vitro* culture of parental and Δgexp15 parasites. Data represent mean ± SD of two independent experiments (n = 60). No significant (ns) difference was observed (Mann–Whitney *U* test). (F) Parental and Δgexp15 parasites were synchronized *in vitro* and mice were intravenously infected. After 1, 22, 25 and 29h post-infection, blood smears were performed and the percentages of rings (in white), trophozoites (in grey) and schizonts (in black) were determined. Data shown represent means ± SD of two independent experiments in duplicate (Tukey’s multiple comparison test, no significant (ns), *** p<0.001, **** p<0.0001 compared to parental at the same timing).

To explore the phenotype(s) of these parasites in more detail, we used two different mouse malaria models, C57BL/6 for experimental cerebral malaria (ECM) and BALB/c for malaria-linked pathologies (severe anemia, hyperparasitemia). The survival was based on euthanizing mice according to criteria described in the Materials and Methods section. When C57BL/6 mice were infected with the parental strain (10^6^ parasites), about 80% succumbed within 6–8 days post-infection from ECM (Fig 3A, Table 1) due to blood-brain barrier disruption as evidenced by Evans blue staining (S4C Fig). In contrast, none of C57BL/6 mice infected with the Δgexp15 parasites exhibited any ECM symptoms and 61% succumbed exclusively to hyperparasitemia between days 20–25 post-infection. In the case of BALB/c mice, as expected, all mice succumbed from hyperparasitemia before day 15 after infection with 10^6^ parental parasites (Fig 3B). Importantly, all BALB/c mice infected with Δgexp15 survived infection with a rapid clearance of all blood parasites after a peak of between days 10 to 12 (Fig 3B and 3C). Given these data, we further tested whether Δgexp15cl1 infected BALB/c mice that survived the infection could be protected from a secondary infection with parental parasites. As depicted in Fig 3D and Table 1, mice challenged with 10^7^ infected red blood cells (iRBC) showed either low parasitemia (<1%) that was quickly cleared or no detectable infection up to 40 days post reinfection, while mice in the control group succumbed to the infection. A similar result was observed with Δgexp15cl2 (Table 1). These results suggest that the infection of BALB/c with Δgexp15 induces a potent protective response against parental parasites.

**Table 1:** Time course of parental and Δgexp15 parasites infection in C57BL/6 and BALB/c mice and protection of BALB/c mice challenged with parental parasites.

Mouse strain	*P*. *berghei* strain		Primo infection dose (No. of iRBCs)^a^	No. of mice surviving from ECM^b^	No. of mice surviving from hyper parasitemia	Challenge dose (No. of iRBCs)^a^^,^^c^	No. of protected mice
C57BL/6	parental	Exp1 Exp2 Exp3	10^6^	1/6 2/6 1/6	0/1 0/2 0/1		
	Δgexp15cl1	Exp1 Exp2 Exp3	10^6^	6/6 6/6 6/6	5/6 1/6 1/6		
BALB/c	parental	Exp1 Exp2	10^6^	5/5 5/5	0/5 0/5		
	Δgexp15cl1	Exp1 Exp2	10^6^	5/5 5/5	5/5 5/5	10^7^	5/5
	Δgexp15cl2	Exp2	10^6^	6/6	6/6	10^7^	6/6
	parental	Exp2	Not infected			10^7^	0/6

^a^ Parasites were inoculated by intraperitoneal injection of infected red blood cells (iRBCs).

^b^ Survival linked to ECM was assessed by specific clinical symptoms at low parasitemia, between 6–8 days post-infection (p.i) and by brains staining using Evans blue.

^c^ Mice were challenged with parental parasites at day 30 post primo-infection.

### Intraerythrocytic development of Δgexp15 parasites

To assess the effects of deficiency of PbGEXP15 expression on parasite growth, we investigated their intraerythrocytic development. First, the number of merozoites per schizont observed *in vitro* did not significantly differ between parental and Δgexp15 parasites (Fig 3E). Thereafter, the growth rate was followed in mice infected intravenously with purified schizonts obtained from overnight cultures of parental and Δgexp15 parasites. At 1h post-infection, as shown in Fig 3F, both parental and Δgexp15 parasites exhibited ~80% rings suggesting a similar ability to invade. After 22h, a slight delay in the maturation of Δgexp15 parasites was observed with 64% of trophozoites and 30% of schizonts versus 41% and 50% respectively in parental parasites. At 25h, the examination of parental parasites showed 63% rings while the Δgexp15 parasites presented 15% rings (p<0.0001). The follow up at 29 h post-infection underscored the delay in transition (85% trophozoites in parental versus 32% in Δgexp15, p<0.0001). These data clearly indicate that the depletion of PbGEXP15 protein delays the intraerythrocytic growth of *P*. *berghei*.

### Role of GEXP15 in transmission to the mosquito

**Fig 4:**
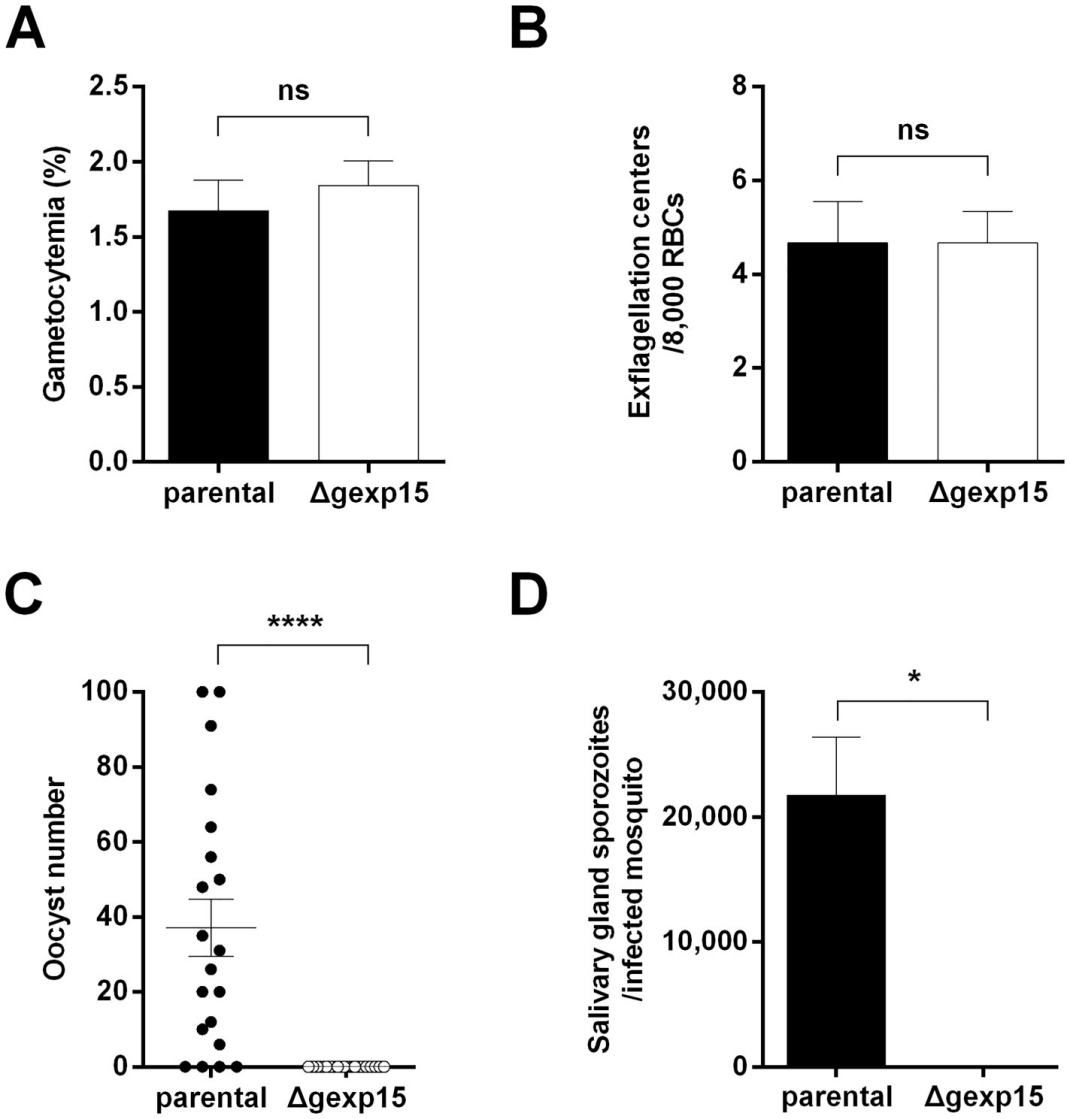
Essentiality of PbGEXP15 in the parasite development in mosquito. (A) Comparison of gametocytemia from parental and Δgexp15 parasites. Mean ± SEM of the number of gametocytes is indicated for two independent experiments (n = 8). No significant difference (ns) was observed (Mann–Whitney *U* test). (B) Comparison of exflagellation from parental and Δgexp15 parasites. Mean ± SEM of the number of exflagellation centers is indicated from two independent experiments (n = 6). No significant difference (ns) was observed (Mann–Whitney *U* test). (C) Comparison of midgut oocysts from parental and Δgexp15 parasites (n = 20). Mosquito midguts were dissected at day 9 and average oocyst number ± SEM is indicated from two independent experiments (Mann–Whitney *U* test, **** p<0.0001). (D) Quantification of sporozoites from mosquitoes infected with parental and Δgexp15 parasites. Salivary glands were dissected at day 18 and sporozoites were manually counted. Bars indicate mean ± SEM of the number of sporozoites per infected mosquito from 4 technical replicates of two independent experiments (Mann–Whitney *U* test, * p<0.05).

Consistent with the first proteomic analysis [19] and this study showing the expression of GEXP15 in gametocytes, we examined whether PbGEXP15 is essential at this stage. Unexpectedly, we noticed that the gametocytemia of Δgexp15 parasites did not differ significantly when compared to parental parasites (Fig 4A) and the deletion seemed to have no effect on the number of exflagellation centers of male gametocytes (Fig 4B). These results indicate that PbGEXP15 did not affect the early male gametocyte development.

In order to examine the role of PbGEXP15 during the mosquito stages and due to the difficulties to obtain reproducible and reliable data from *in vitro* ookinete conversion assays with the pG230 line that showed a very low conversion efficiency to ookinetes [36], we used parental or Δgexp15 infected mice to feed *Anopheles stephensi* mosquitoes. Two independent experiments were performed and the dissection of the mosquito midguts at day 9 confirmed that 80% of blood meals were positive with parental parasites (Fig 4C). Interestingly, in contrast to parental parasites, Δgexp15 parasites failed to initiate the formation of oocysts. This result was confirmed by the lack of Δgexp15 sporozoites in the salivary glands observed at day 18 whereas 22,000 sporozoites, on average, were detected with parental parasites (Fig 4D). We conclude that PbGEXP15 is essential for parasite development in the mosquito.

### Quantitative proteomic and phosphoproteomic analyses of Δgexp15 schizonts

Previous studies showed that phosphatase inhibitors, comprising those acting on PP1, are toxic to cells and that the uncontrolled activity of the catalytic phosphatase subunits could cause apoptotic cell death [37]. Given the capacity of PbGEXP15 to interact with and regulate the dephosphorylation activity of PP1, we explored whether PbGEXP15 depletion could change the global phospho-proteomic patterns of Δgexp15 parasites. To investigate this, parental and Δgexp15cl1 schizonts were compared to assess proteomic and phosphoproteomic profiles. The quantitative experiments were performed on four biological replicates and three technical replicates.

**Fig 5:**
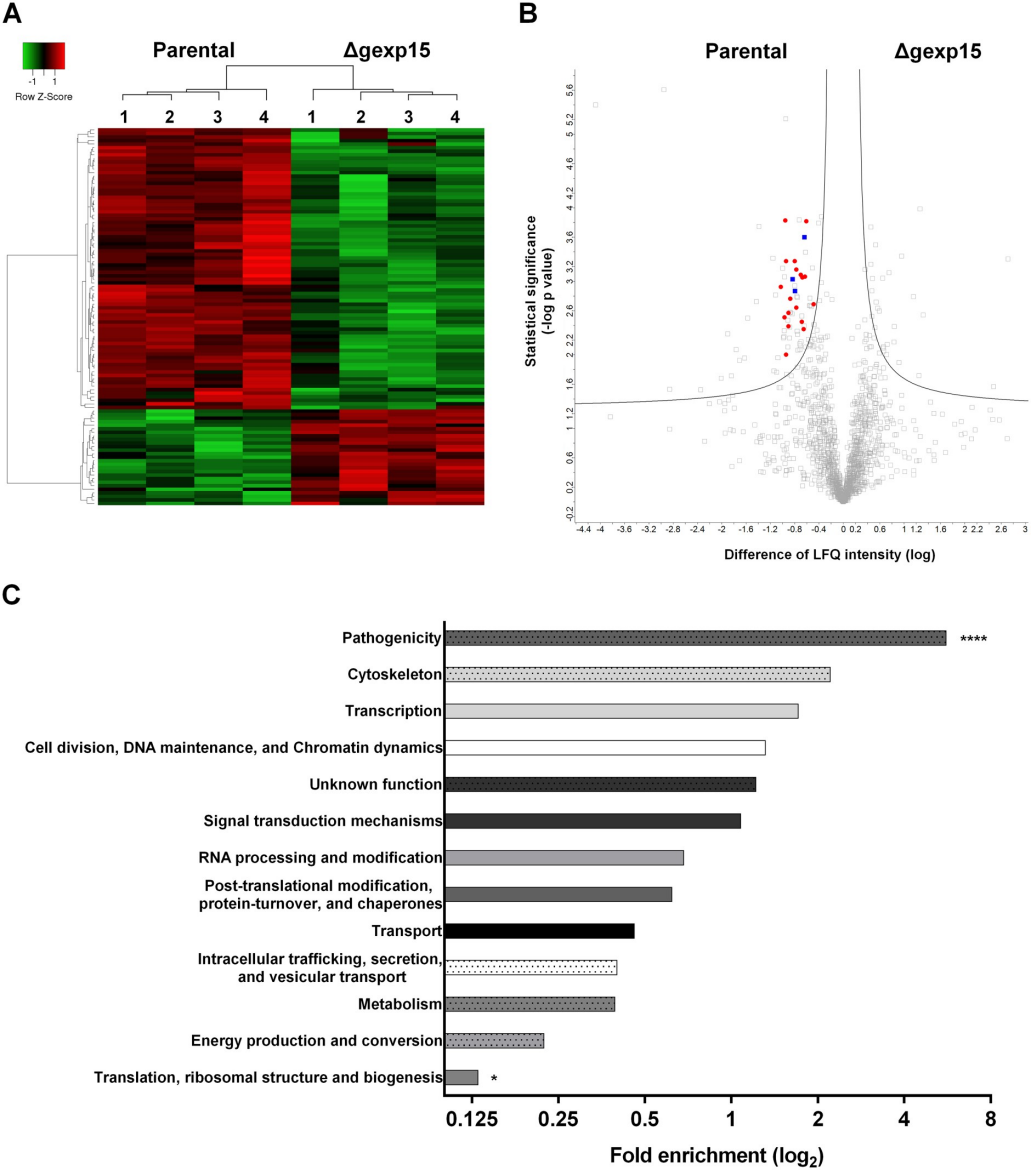
Differential proteome of parental and Δgexp15 schizonts in *P*. *berghei*. (A) Hierarchical clustering and heatmap of proteins with a significant differential abundance in parental and Δgexp15 schizonts (FDR<0.05, S0 = 0.1). (B) Volcano plot representation of proteins quantified by mass spectrometry in parental and Δgexp15 schizonts (FDR<0.05, S0 = 0.1). AP2 transcription factors are indicated as blue squares and proteins involved in the pathogenicity as red dots. (C) Functional enrichment of parental and Δgexp15 schizonts proteomic profiles. Fold enrichment was performed on the 106 filtered proteins relative to all *P*. *berghei* proteins detected in the proteome. The x-axis represents the fold enrichment (log_2_) for the indicated function (Hypergeometric test, ** p<0.01, **** p<0.0001).

First, for the whole proteome analysis, we identified 2188 *Plasmodium* proteins across samples corresponding to 43% of the predicted proteome of *P*. *berghei*. We retained 1484 *Plasmodium* proteins that were reliably quantified in four biological replicates in at least one group (S4 Table). In this global proteome, PbPP1 was detected and no difference was observed between levels in parental and Δgexp15 parasites. We next focused on proteins with significant changes in parental and Δgexp15 proteomes (FDR<0.05). The results indicate a total of 106 proteins (accounting for ~ 7% of the total proteins identified) whose abundance is significantly altered, of which 27 and 79 showed a significant increase and decrease respectively in comparison to parental parasites (Fig 5A and 5B and S4 Table). An enrichment analysis of the biological processes was then performed for these proteins versus the global proteome (Fig 5C). Interestingly, 16 proteins playing a role in the pathogenicity, such as RONs and MSPs, were detected with a significant decrease in the Δgexp15 parasites and correspond to the highest significant enrichment (5.6-fold, p<0.001) (Fig 5B and 5C). We also noticed an under-representation of proteins involved in translation (0.13-fold, p<0.01) while 3 AP2 transcription factors were enriched among the up-regulated proteins (5.25-fold, p<0.05) (Fig 5B and 5C and S4 Table). Of note, many proteins involved in these three biological processes have already been described to interact with PP1 (this study and [17,32,38]). In total, 12% (13/106) of the altered proteins, such as AMA1, RON-2, -4 and -5, are detected in the *Plasmodium* interactomes of PP1 (S5A Fig and S4 Table). Collectively, these data indicate that the knock-out of *pbgexp15* impacted the expression of several PP1 partners and would tend to confirm the commonality of signaling pathway(s) between GEXP15 and PP1.

**Fig 6:**
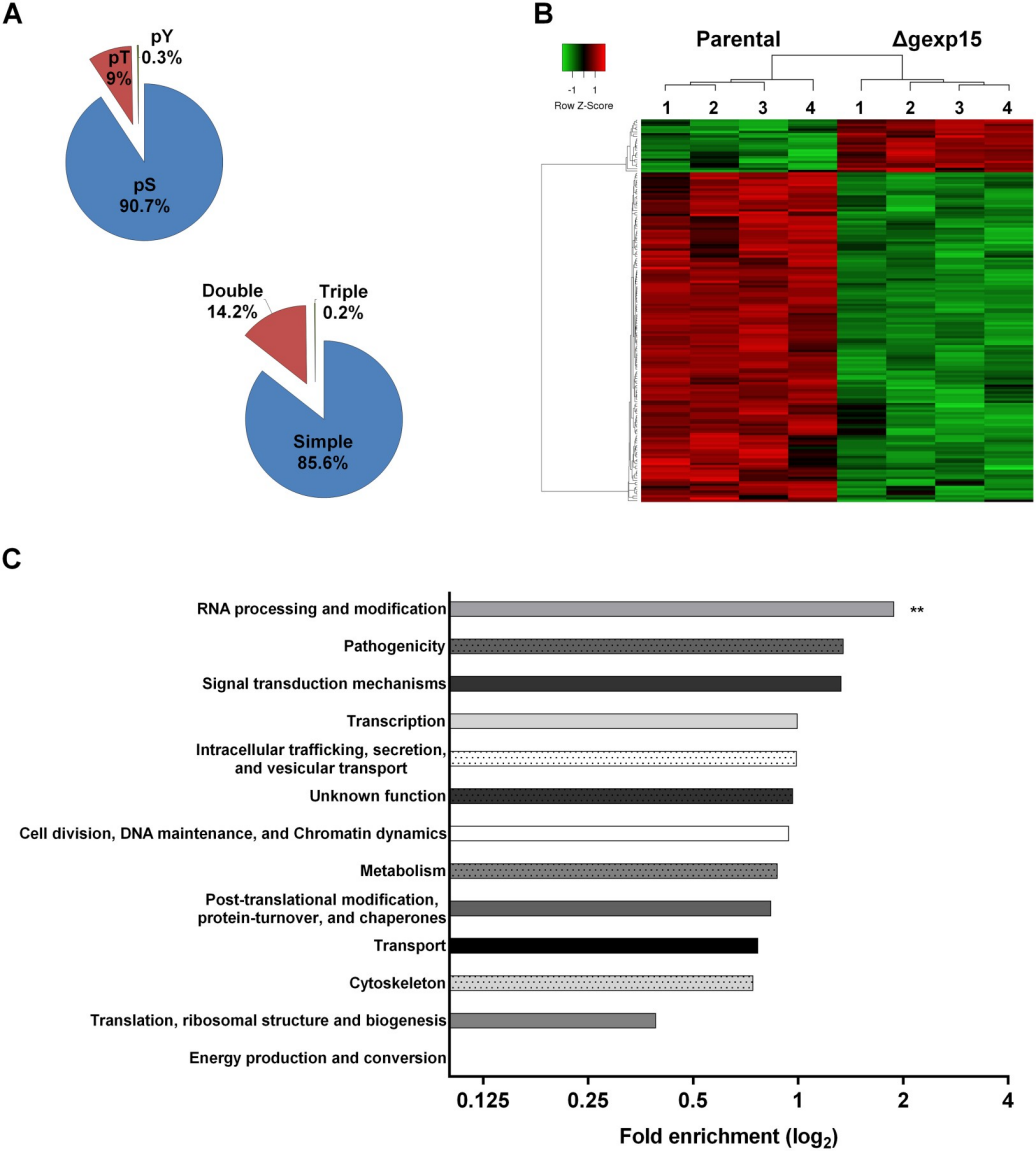
Differential phosphoproteome of parental and Δgexp15 schizonts in *P*. *berghei*. (A) Distribution of phospho-Ser (pS), phospho-Thr (pT), and phospho-Tyr (pY) residues and multiplicity of phosphosites detected in schizonts (n = 2460). (B) Hierarchical clustering and heatmap of phosphosites with a significant differential in parental and Δgexp15 schizonts (FDR<0.01, S0 = 0.1). (C) Functional enrichment of parental and Δgexp15 schizont phosphoproteomic profiles. Fold enrichment was performed on the 166 filtered phosphosites relative to all *P*. *berghei* phosphosites detected in the phosphoproteome. The x-axis represents the fold enrichment (log_2_) for the indicated function (Hypergeometric test, ** p<0.01).

Next, we explored the phosphoproteome of parental and Δgexp15 parasites. In total 2460 different phosphorylation sites of *P*. *berghei* were identified and quantified belonging to 780 *Plasmodium* proteins (Fig 6A and S5 Table). We observed significant changes for 166 phosphopeptides corresponding to 118 proteins (FDR<0.01) (Fig 6B and S5 Table). Levels of phosphorylation of most of the phosphosites (143 phosphosites) were lower in Δgexp15 when compared to the phosphoproteome of the parental parasites. The analysis showed a significant enrichment in proteins acting on RNA metabolism (1.9-fold, p<0.01) with 11 out of 12 phosphosites hypophosphorylated in Δgexp15 parasites (Fig 6C). We also noticed the hypophosphorylation of 8 proteins engaged in transcriptional regulation (including AP2 transcription factors), 5 proteins playing a role in post-translational modifications/chaperones and 4 proteins involved in trafficking (S5 Table). A smaller set of 19 proteins was found to be hyperphosphorylated in the Δgexp15 parasites but most of these proteins have an unknown function. We also observed that overall 18% (21/118) of the phosphoproteins showing significant changes in phosphorylation were previously reported in the *Plasmodium* interactomes of PP1 suggesting that the phenotypes observed above could be due to a deregulation of the PbPP1 activity (S5A Fig).

### Quantitative proteomic and phosphoproteomic analyses of Δgexp15 gametocytes

To explore more deeply the role and the essentiality of GEXP15 in sexual and mosquito stages, we purified parental and Δgexp15cl1 gametocytes for proteomic and phosphoproteomic studies. The quantitative experiments were performed on three biological replicates and three technical replicates.

**Fig 7:**
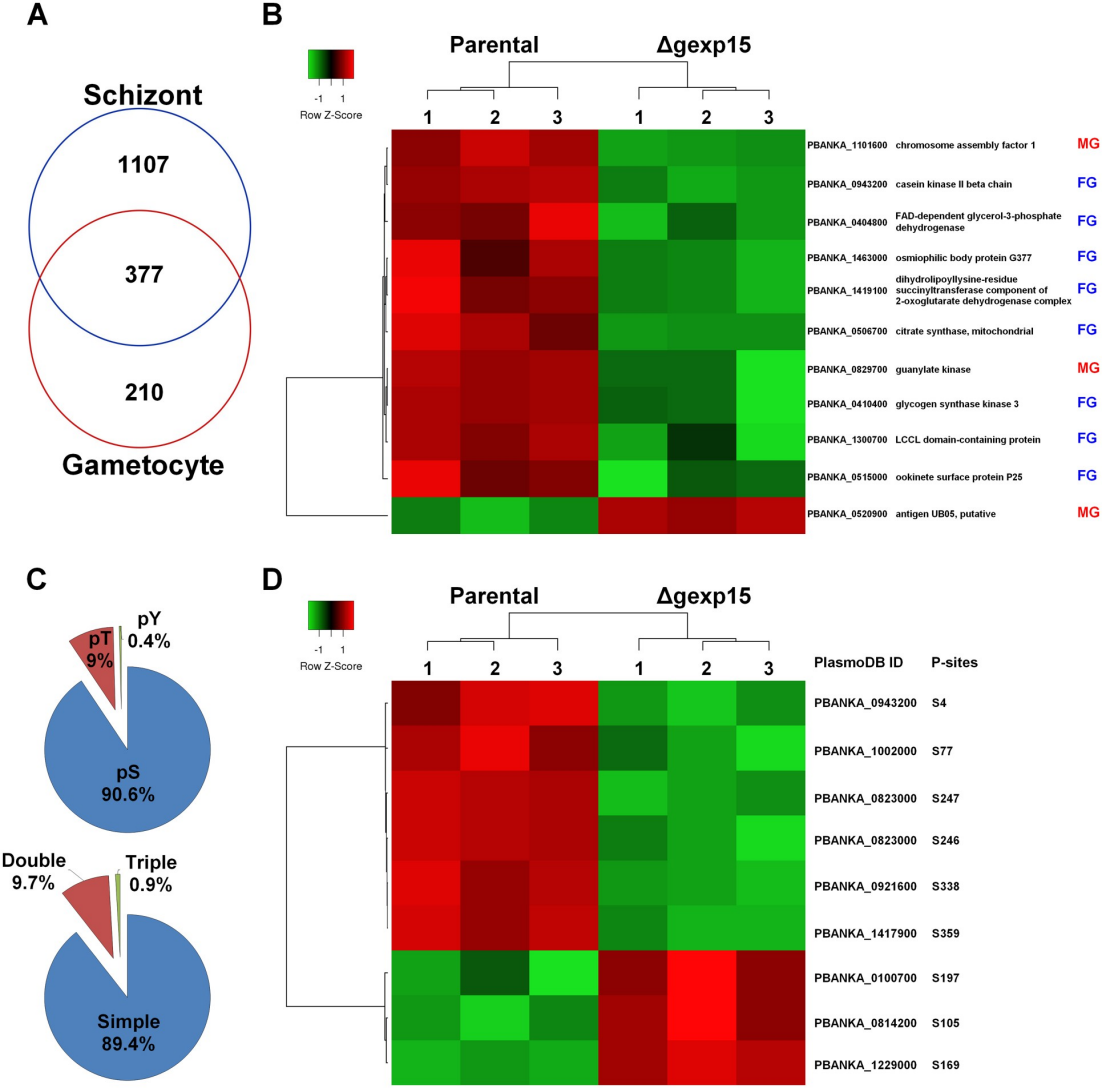
Differential proteome and phosphoproteome of parental and Δgexp15 gametocytes in *P*. *berghei*. (A) Venn diagram of proteins detected in the proteomes of schizonts and gametocytes. (B) Hierarchical clustering and heatmap of proteins with a significant differential abundance in parental and Δgexp15 gametocytes (FDR<0.05, S0 = 0.1). The sex enrichment in gametocyte stage is indicated for each protein. MG: male gametocyte, FG: female gametocyte [40–42]. (C) Distribution of phospho-Ser (pS), phospho-Thr (pT), and phospho-Tyr (pY) residues and multiplicity of phosphosites detected in gametocytes (n = 444). (D) Hierarchical clustering and heatmap of phosphosites with a significant differential in parental and Δgexp15 gametocytes (FDR<0.01, S0 = 0.1). The position and the residue of the phosphosites are indicated.

Firstly, we determined the gametocyte proteome and identified 587 *Plasmodium* proteins that were reliably quantified in three biological replicates in at least one group (S6 Table). As depicted in Fig 7A, 64% of these proteins are also detected in the schizont proteome, a percentage similar to previous overlaps in *P*. *berghei* (50%) and *P*. *falciparum* (59%) [39,40]. Significant changes in parental and Δgexp15 proteomes (FDR<0.05) were observed for 11 proteins, with a significant decrease in abundance for 10 proteins in mutant parasites (Fig 7B and S6 Table). One of these proteins, PbGSK3 (PBANKA_0410400) that decreased significantly in Δgexp15 gametocytes and for which commercial antisera were available was tested by western blot experiments in two independent biological samples. Results indicated a drastic decrease of this protein in Δgexp15 gametocytes when compared to the parental strain, validating the proteomic analysis (S5B and S5C Fig).

Interestingly, 8 proteins out of the 11 have been described being enriched in female gametocytes and the others 3 in male gametocytes [39–42]. Among these proteins, we detected G377 (PBANKA_1463000) which is localized in the osmiophilic bodies of female gametocytes and plays a role in the formation of these organelles and in the induction of gametogenesis with a delay in female gamete egress [43,44]. Furthermore, diverse studies have demonstrated that P25 (PBANKA_0515000) and Lap2 (PBANKA_1300700) play important roles in parasite transmission by mosquitoes and more precisely in the ookinete and oocyst stages [45–48]. Altogether, these data confirmed the phenotypic analyses observed and suggested that the deletion of *pbgexp15* impacted key proteins in mosquito transmission. Concerning the phosphoproteome of parental and Δgexp15 parasites, we identified 444 different phosphorylation sites of *P*. *berghei* belonging to 278 proteins and we observed significant changes for only 9 phosphopeptides corresponding to 8 proteins (FDR<0.01) (Fig 7C and 7D and S7 Table). Unlike the proteome, these proteins did not seem to exhibit features linked to sexual differentiation similar to those shown above. Only two proteins are clearly described to be enriched in male and one in female gametocytes (S7 Table).

## Discussion

The functional diversity of the PP1 catalytic subunit, an essential phosphatase enzyme, is now clearly attributable to more than 200 regulators that have been described in diverse eukaryotic organisms [8,9]. To date, only four conserved regulators of PP1 have been identified and characterized in *P*. *falciparum* [10–12,15]. A more recent study, using an Y2H screening to examine the global PP1 interactome in *P*. *falciparum*, identified GEXP15 as a potent regulatory partner of PP1 [17]. Here we confirmed the direct interaction of PbGEXP15 with PP1 and demonstrated its capacity to control the phosphatase activity. These results extend our previous data showing the capacity of conserved regulators to affect PP1 activity to a specific protein expressed by *Plasmodium*. Structure-activity studies indicate a major contribution of the well-known RVxF consensus binding motif to the function of GEXP15. Moreover, a short N-terminal region of GEXP15 was able to bind PP1 and to increase its phosphatase activity in a similar manner to that observed with the full length protein. The mutation of the RVxF motif, present in this N-terminal region, completely abolished this regulation. These data suggest that this region seems to carry the regulatory function of GEXP15 on PP1 activity.

To further explore the functional role of GEXP15, we examined the impact of its deletion in the rodent malaria parasite *P*. *berghei*. Phenotypic analyses of these deficient parasites revealed a drastic effect on their development both in mice and mosquitoes. Indeed, while BALB/c mice infected with parental parasites succumbed to infection from hyperparasitemia, they were able to efficiently clear Δgexp15 parasites. This could be linked at least in part to the retarded multiplication that we observed. Further, surviving mice exhibited a protection against a secondary challenge by parental parasites, suggestive of a role of these deficient parasites in inducing protective responses.

When C57BL/6 mice susceptible to ECM were tested, Δgexp15 parasites were found to be unable to induce ECM. Investigations on ECM in the mouse model indicated that it is a complex process involving both the parasite and the host molecules including proinflammatory cytokines [49–52]. However, when the outcomes of infections by Δgexp15 parasites of C57BL/6 and BALB/c mice (prototypical Th1 and Th2-type strains respectively) were compared, we observed a higher rate of mortality in C57BL/6 (ECM model) due to hyperparasitemia than in BALB/c (hyperparasitemia model), suggesting a difference in the immune responses raised by these hosts towards the infection with Δgexp15 parasites. Earlier studies mainly focused on phenotypic analyses have shown similar data obtained with targeted disruption of hmgb2 described as a pro-inflammatory protein [53], MSP7 involved in invasion of erythrocytes [54] or plasmepsin-4 contributing to hemoglobin digestion [55]. Although, the parasite derived molecules triggering host responses are still largely unknown, the use of Δgexp15 parasites might contribute for a better understanding of the protective mechanisms against ECM.

Of note, the essentiality of GEXP15 in the blood stages was further supported by a recent study using a piggyBac transposon inserted randomly in *P*. *falciparum* genome [56] in which they did not obtain viable parasites with a disrupted *pfgexp15* gene despite the presence of 35 potential insertion sites.

For an in-depth dissection of the biological functions of GEXP15, we performed quantitative proteomic and phosphoproteomic analyses. To the best of our knowledge, our proteomic study is the first in which a PP1 partner has been suppressed in *Plasmodium*.

Of particular interest are low abundance proteins in schizonts that are members of the AP2 transcription factor family, which play a role in the regulation of gene expression, and invasion proteins including RONs and AMA1. The low abundance of three AP2 transcription factors, that have been suggested to be essential [57,58], could explain the general down-regulation observed for the proteins whose expression varies in Δgexp15 parasites. These results highlight the role played by GEXP15 and could explain the attenuated virulence of Δgexp15 parasites in blood stages.

In our global phosphoproteomic analysis in schizonts, the data indicate the hyperphosphorylation of 19 proteins in Δgexp15 when compared to parental parasites. This could be expected as the lack of GEXP15, an activator of PP1 *in vitro*, may lead to a decrease in PP1 activity and consequently an increase of phosphorylation of target proteins. Should this be the case, these hyperphosphorylated proteins could be considered as potential substrates of the complex PP1-GEXP15. Interestingly, the RON2 protein, present in lower abundance in Δgexp15 parasites, was found to be hyperphosphorylated, possibly attenuating its known function in invasion [59–63]. However, synchronized Δgexp15 parasites did not show any delay in the first invasive cycles, suggesting a functional overlap and/or compensation between proteins involved in invasion [64] or that the induced defect did not attain a sufficient threshold to interrupt the invasion at least during the first cycles. On the other hand, the data suggest an unanticipated role of GEXP15 in the phosphorylation process. Indeed, we observed a drop in the phosphorylation levels of 100 proteins when compared to controls. These data could be explained either by free and uncontrolled PP1 capable of non-specifically dephosphorylating many and diverse substrates in the absence of GEXP15 and/or by an inhibitory role of GEXP15 on PP1 activity in *Plasmodium*. This latter possibility cannot be excluded as *in vitro* experiments, which showed a positive effect of GEXP15 on PP1 activity, were performed with the non-natural pNPP substrate. In addition, any post-translational modifications of GEXP15 *in vivo* could affect its function, which may be different from that observed with the recombinant protein. In this context, it has been shown that the phosphorylation status of Inhibitor-1, a well-known regulator of human PP1, differentially alters its function [65–67].

Concerning the proteome in gametocyte stages, we detected 11 proteins whose expression varies in Δgexp15 parasites. Among these proteins, eight, described to be overexpressed in female gametocytes, are present at lower levels in these deficient parasites. These data along with the above observations indicate that the lack of GEXP15 impacts neither the number of gametocytes nor the exflagellation of male gametocytes and strongly suggest that GEXP15 could function downstream in female gamete formation/egress/fertility or zygote-to-ookinete development. Supporting this is the under-abundance of the G377 protein in Δgexp15 that has been reported as a key actor in female egress/emergence (43, 44). Additional proteins could also contribute to the observed phenotype include Lap2, demonstrated as being important in the mosquito transmission [47,48] or AP2-O2 that was hypophosphorylated on 2 serines in Δgexp15 schizonts compared to the parental parasites. This latter transcription factor is described as being crucial in the development of ookinetes and oocysts in *P*. *berghei* [58] while in *P*. *yoelii*, a knock-out of AP2-O2 did not seem to affect the gametocytes and ookinetes but only the number of oocysts and sporozoites [68]. In this study, unfortunately, the pG230 line used to generate Δgexp15 parasites exhibits very low conversion efficiency *in vitro*, hampering these studies. Of note, transfections of two other *P*. *berghei* strains did not allow the generation of viable knock-out or stable inducible knock-down parasites. Whatever the explanation, it is clear that the depletion of GEXP15 led to a complete abolition of oocyte/sporozoite formation *in vivo*. Taken together, our observations suggest that while the lack of GEXP15 expression could be transiently compensated in intraerythrocytic growth in the blood, this compensation seems to be insufficient with a high fitness cost in the mosquito.

Interestingly, an earlier study suggested that GEXP15 was a potential orthologue of human CD2BP2 (CD2 Cytoplasmic Tail Binding Protein 2) [69]. CD2BP2 has been described to interact with splicing factors and PP1 through a GYF domain [34] and an RVxF motif respectively [35]. The sequence analysis of PbGEXP15 reveals only 14% identity with HsCD2BP2, but it presents a GYF like-domain, even if this does not match perfectly the consensus sequence [70]. However, although the IP/MS of PbGEXP15-mCherry demonstrated that several splicing factors are potential partners of GEXP15, its contribution in the splicing function in *Plasmodium* requires further investigation in the future.

In conclusion, the results shown here indicate that the viability of parasites in the absence of GEXP15 expression is accompanied by major alterations that could contribute to the avirulent phenotype of these parasites and their incapacity to produce oocysts. These alterations could affect spliceosome and proteasome pathways along with extensive changes to the phosphorylation patterns of Δgexp15 parasites that may be linked to an uncontrolled PP1, capable of dephosphorylating inappropriate substrates. Additional studies are required to examine each novel aspect of the phenotypes of these Δgexp15 parasites and the potential alteration of the RNA splicing pathway.

## Materials and methods

### Plasmids

Plasmids pGADT7/pGBKT7, pETDuet-1 and pGEX4T3 were purchased from Clontech, Novagen and GE Healthcare Life Sciences. The plasmids pBS-DHFR and pL1886 were kindly provided by Drs R. Tewari and B. Franke-Fayard respectively. The primers used are described in S8 Table.

### Ethics statement

Mice were housed in an Animal Biosafety Level 2 facility at the Institut Pasteur de Lille and maintained in accordance with the French National Guidelines for Use of Animals for Scientific Purposes which is also in line with EU Directive 2010/63/EU. Experimental protocols performed in this study were reviewed and approved by the Comité d’Ethique C2EA-75 en Expérimentation Animale Nord-Pas de Calais-France (project number: 00527.04).

### Animals

Infections and antiserum production were performed in CD1 male mice (30g) (Charles River). BALB/c (10 weeks) and C57BL/6 (4-5weeks) male mice (Janvier Labs) were sorted randomly into groups of 5–6 animals and used for hyperparasitemia and ECM comparison between parental and Δgexp15 parasites. The duration of experiments was strictly limited and constant monitoring of infected mice was carried out. When parasitemia was about 60% accompanied with weight body loss, mice were euthanized by CO_2_ inhalation. Mice susceptible to ECM were euthanized by CO_2_ inhalation if they displayed paralysis, convulsions/fits or coma. For the disruption of blood brain barrier, 100μl of 2% Evans blue dye in PBS were injected intravenously in C57BL/6 infected mice at day 6 p.i. Mice were then euthanized by CO_2_ inhalation 1h post-injection and brains were recovered.

### Yeast two-hybrid assays

PbGEXP15 _4–590_, PbGEXP15 _4–178_, PbGEXP15 _446–596_ were amplified by PCR on *P*. *berghei* gDNA with primers p1-p2, p1-p3, p4-p5 respectively (S8 Table) and cloned into the pGADT7 vector (Clontech) using the In-Fusion HD Cloning system (Clontech) according to the manufacturer’s instructions. Gal4-DBD-PfPP1c and PfPP1c F255A/F256A were previously cloned [12,23]. PbGEXP15 _4–178 KKKKKAQA_ was obtained by PCR-based site directed mutagenesis with Isis DNA polymerase (MP Biomedicals) and pGADT7-PbGEXP15 _4–178_ as template and primers p6-p7. pGADT7 and pGBKT7 constructs were transformed into Y2H Gold and Y187 yeast strains (Clontech) respectively, and the yeasts were spread on Synthetic Defined agar medium lacking leucine (SD-L) or lacking tryptophane (SD-W) respectively and grown at 30°C for 3–5 days. Different mating experiments were performed and spread on selective media SD-LW. They were restreaked on more stringent media SD-LWH and SD-LWHA (L: Leucine, W: Tryptophan, H: Histidine, A: Adenine) after dilutions at 1:1, 1:25, 1:50. Diploids were incubated for 4–6 days at 30°C. Empty vectors pGADT7 or pGBKT7 and pGBKT7-Laminin were used as negative controls.

In order to check *Plasmodium* gene expression in yeast, RT-PCRs were performed. Total RNA was isolated from cultured yeasts (OD = 0.5) after flash freezing and using TRIzol Reagent (Thermo Fisher Scientific) with glass beads for 45 min at 65°C with occasional vortexing. RNA (5 μg) was treated with DNAse I (Thermo Fisher Scientific) and DNA contamination was checked using an Agilent 2100 Bioanalyzer and by RT-PCR on intronic gene of yeast *tub1*. cDNA was synthesized using SuperScript III First-Strand Synthesis SuperMix (Thermo Fisher Scientific) according to the manufacturer’s instructions. Amplification of transcripts was carried out by PCR using the Advantage 2 Polymerase Mix (Clontech) and the following primers (S8 Table): p13-p14 for TUB1, p11-p12 for Gal4-DBD-PfPP1c and Gal4-DBD-PfPP1c F255A F256A, p8-p9 for Gal4-AD-PbGEXP15 _4–590_, Gal4-AD-PbGEXP15 _4–178_ and Gal4-AD-PbGEXP15 _4–178 KKKKKAQA_, and p8-p10 for Gal4-AD-PbGEXP15 _446–596_.

### Recombinant protein expression and antiserum production

The coding regions of PbGEXP15 _4–590_ and PbGEXP15 _4–178_ were obtained by PCR with the primers p15-p16 and p15-p17 respectively (S8 Table) and cloned into pETDuet-1 (Novagen) using the In-Fusion HD Cloning system (Clontech). PbGEXP15 _4–178 KKKKKAQA_ was obtained by PCR-based site directed mutagenesis with Isis DNA polymerase (MP Biomedicals), pETDuet-1-PbGEXP15 _4–178_ as template and primers p6-p7.

GST, GST-PfPP1c and PfPP1c were produced as previously described [11,15]. All recombinant GEXP15 expressions were carried out in One Shot^®^ BL21 Star^™^ (DE3) Chemically Competent *E*. *coli* cells (Life Technologies) in the presence of 0.5 mM IPTG at 16°C overnight. Cells were harvested in non-denaturing buffer (20 mM Tris, 500 mM NaCl, 20 mM imidazole and protease inhibitor cocktail (Roche), pH 7.5) followed by sonication and ultracentrifugation. Pellets were resuspended and centrifuged in denaturing buffer (20 mM Tris, 500 mM NaCl, 6 M guanidine, 20 mM imidazole and protease inhibitor cocktail (Roche), pH 7.5). Ni^2+^-NTA agarose beads (Macherey Nagel) were used to purify the recombinant proteins as previously described [12]. SDS-polyacrylamide gels were blotted onto nitrocellulose and probed with anti-His antibody (1:2000 dilution) (Qiagen) followed by HRP-labeled anti-mouse IgG (1:50000 dilution). Chemiluminescence detection with SuperSignal^™^ West Dura Extended Duration Substrate (Life Technologies) was carried out. Recombinant proteins were quantified with Pierce^™^ BCA Protein Assay Kit (Life Technologies).

The purified PbGEXP15 _4–178_ was used to produce antisera as previously described [12] in CD1 mice.

### GST pull-down assays

Glutathione-Sepharose beads (Sigma-Aldrich) coupled with GST-PfPP1c were incubated overnight at 4°C with 2 μg of PbGEXP15 _4–590_, PbGEXP15 _4–178_ or PbGEXP15 _4–178 KKKKKAQA_ in 20 mM Tris, 150 or 500 mM NaCl, 0.2 mM EDTA, 20 mM HEPES, 1 mM MnCl_2_, 1 mM DTT, 0.1% Triton X-100, 10% glycerol, protease inhibitor cocktail (Roche) and pH 7.5. After 5 washes of the beads with the same buffer, proteins bound to the beads were analyzed by 4–20% SDS–PAGE followed by immunoblotting with anti-His (1:2000) or anti-GST mAb (1:2000) (Invitrogen) as described above.

### Effect of GEXP15 on PfPP1 activity

The role of GEXP15 on the activity of PfPP1c was investigated using the p-nitrophenyl phosphate (pNPP) assay. Different amounts of PbGEXP15 _4–590_, PbGEXP15 _4–178_ and PbGEXP15 _4–178 KKKKKAQA_ were preincubated with 40 pmol of PfPP1c for 30 min at 37°C. The enzymatic reaction was initiated by the addition of pNPP substrat (Sigma-Aldrich) to the reaction medium and the absorbance was measured at 405 nm (Thermo Scientifc Multiskan FC). The lack of phosphatase activity of recombinant GEXP15 alone was checked according to the described procedure in the absence of PP1. Two independent experiments were carried out in duplicate.

### Generation and analysis of *P*. *berghei* transgenic parasites

In order to tag PbPP1c with mCherry, *pbpp1c* was amplified with primers p18-p19 (1324 bp) (S8 Table). The insert was subcloned into pL1886 plasmid. The construct was linearized by Tth111I before transfection. The same plasmid was used for PbGEXP15-mCherry. The 3’ region of *pbgexp15* was amplified with primers p22-p25 and p24-p23 (1332 bp) and a silent mutation was introduced by PCR-based mutagenesis in order to obtain a BsmI site, used to linearize the plasmid.

For the knock-out of *pbgexp15*, PCR amplifications were generated with the 5’ and 3’ UTR regions with primers p26-p27 (847 bp), p28-p29 (695 bp) and *P*. *berghei* gDNA as template. The inserts were subcloned into pBS-DHFR plasmid [71] and the construct was linearized by XbaI-ApaI before transfection.

Linearized pL1886 plasmids and pBS-DHFR plasmid were transfected by electroporation as previously described [72] in *P*. *berghei* ANKA GFP line [24] and pG230 line [36] respectively, kindly provided by Drs O. Silvie and N. Philip. Transfected parasites were inoculated in CD1 mice and positively selected by pyrimethamine in the drinking water, 30h after transfection [72].

Parasitized erythrocytes were lysed with Red blood cell Lysing buffer (Sigma-Aldrich) followed by the use of KAPA Express Extract kit (KAPA BioSystems) to extract DNA (manufacturer’s instructions). Primers p20-p21 and p15-p21 were used to genotype *pbpp1c*-mCherry and *pbgexp15*-mCherry respectively (S8 Table). Deletion of *pbgexp15* was verified by diagnostic PCR using primers p30-p31 and p32-p33 and positive *pbgexp15* knock-out parasites were cloned via intraperitoneal injection in CD1 mice.

### Immunofluorescence assays

Blood from mice infected with PbPP1-mCherry or PbGEXP15-mCherry parasites was fixed with 4% paraformaldehyde and 0.0075% glutaraldehyde for 10 min at 4°C. After PBS washing, cells were sedimented on Poly-L-lysine coated coverslips overnight then permeabilized and saturated with PBS, 0.5% Triton X-100 and 1% BSA for 30min. Anti-RFP pAb (MBL, PM005) was diluted 1:500 in PBS BSA 1% and applied for 1h at 37°C. Coverslips were washed with PBS and incubated with Goat anti-Rabbit IgG (H+L) Cross-Adsorbed, Alexa Fluor 594 (Invitrogen, A11012) in PBS BSA 1% at 1:1000 in addition to DAPI (1μg/ml) for 1h at 37°C. The coverslips were mounted in Mowiol and confocal imaging was performed with an LSM880 microscope (Zeiss). Images were treated with ImageJ.

### Purification of schizonts and gametocytes in *P*. *berghei*

To obtain schizonts, blood from infected mice was incubated 20h at 37°C in RPMI1640 culture medium supplemented with 0.4% AlbuMAX^™^ II Lipid-Rich BSA (Life technologies), then schizonts were purified on a 55% Nycodenz gradient. Gametocytes purification was performed as previously described [73]. Briefly, CD1 mice were treated with phenylhydrazine by the intraperitoneal route (200μl, 6mg/ml, Sigma-Aldrich) 2 days pre-infection then treated with sulfadiazine (20mg/ml in drinking water, Sigma-Aldrich) 3 days post-infection. At day 5 post-infection, blood was collected by cardiac puncture and gametocytes purified on a 48% Nycodenz column in coelenterazine buffer. Purifications were higher than 95% for schizonts and gametocytes.

### Immunoprecipitation and mass-spectrometry

Purified schizonts or gametocytes of PbPP1-HA, PbPP1-mCherry, PbGEXP15-mCherry and parental wild-type parasites used as control, were suspended in 50 mM Tris, 0.5% Triton X-100 and protease inhibitor cocktail (Roche), pH 8. After 10 freeze-thaw cycles and sonication, soluble fractions were obtained after repeated centrifugations at 13000 rpm at 4°C. Anti-HA agarose beads (Life Technologies) or RFP-Trap^®^_A beads (Chromotek) were mixed overnight at 4°C with parasite soluble extracts in 20 mM Tris, 150 mM NaCl, 0.5% Triton X-100 and protease inhibitor cocktail (Roche), pH 7.5. Beads were washed and elution was performed in Laemmli buffer. Then after 3 min at 95°C, samples were loaded on a 4–20% SDS-PAGE for western blot or mass spectrometry analyses. Western blots were carried out as described above and probed with anti-RFP pAb (1:1000, MBL) followed by goat anti-rabbit IgG-HRP (1:20000, Sigma-Aldrich). Then, the membrane was stripped and probed with mouse sera anti-GEXP15 (1:100) followed by Mouse TrueBlot^®^ Ultra: Anti-Mouse Ig HRP (1:2000, eBioscience). For the Mass-spectrometry analysis, electrophoretic migration, tryptic digestion and nanoLC-MSMS analysis were performed as previously described [74]. Raw data collected during nanoLC-MS/MS analyses were processed and converted into *.mgf peak list format with Proteome Discoverer 1.4 (Thermo Fisher Scientific). MS/MS data were interpreted using the search engine Mascot (version 2.4.0, Matrix Science, London, UK) installed on a local server. Searches were performed with a tolerance on mass measurement of 0.2 Da for precursor and 0.2 Da for fragment ions, against a composite target decoy database (2*22,202 total entries) built with *Mus musculus* Uniprot database (10090–17,008 entries), *Plasmodium berghei* PlasmoDB database (Release 41.0–5 December 2018–5,076 entries) fused with the sequences of PbPP1-HA or PbGEXP15-mCherry, recombinant trypsin and a list of classical contaminants (118 entries). Cysteine carbamidomethylation, methionine oxidation, protein N-terminal acetylation, and cysteine propionamidation were searched as variable modifications. Up to one trypsin missed cleavage was allowed. For each sample, peptides were filtered out according to the cut-off set for proteins hits with one or more peptides longer than nine residues. Ion and identity score were fixed to obtain a 1% false positive rate.

### Asexual and sexual development assays

In order to evaluate the number of merozoites per schizont, purified schizonts from *P*. *berghei* cultures were fixed in 4% paraformaldehyde and 0.0075% glutaraldehyde for 10 min at 4°C then incubated 30 min with DAPI. The nuclei were counted with Leica Leitz DMRB fluorescence microscope. To measure growth rate, purified mature schizonts were intravenously injected in CD1 mice. Smears were performed at 1h, 22h, 25h and 29h post-infection and rings, trophozoites and schizonts were counted under the microscope after Giemsa staining.

For sexual development, the gametocytemia was determined by microscopy. Exflagellation was assessed after 10–12 min of incubation at 21°C in RPMI 1640 with 25 mM HEPES and 10% fetal calf serum, pH 8 [75]. The exflagellation centers were counted under slide-coverslip by microscopy using a 40x objective.

### Mosquito transmission

*Anopheles stephensi* mosquitoes were maintained at the insectarium of the Institut Pasteur de Lille. They are reared at 19°C and 75–80% humidity under 12/12 hour light/dark cycle. Female mosquitoes (4 to 6 days) were fed on anaesthetized infected CD1 mice once gametocytes had been observed. The presence of oocysts in the midgut was checked at day 9 post blood meal and dissection of salivary glands was assessed at day 18. Fifteen salivary glands were pooled and homogenized per technical replicate. Sporozoite counts were determined with a Kova slide. The experiments were performed twice independently.

### Sample preparation and mass spectrometry

*P*. *berghei* schizonts or gametocytes were purified as described above and treated with 0.15% saponin to avoid host contamination. Soluble proteins were extracted in RIPA buffer (Thermo Fisher Scientific), Halt^™^ Protease and Phosphatase Inhibitor Cocktail (Thermo Fisher Scientific) and DNase I (Thermo Fisher Scientific). Proteins were quantified with the Pierce^™^ BCA Protein Assay Kit (Life Technologies). 82 μg of proteins for the schizonts and 100 μg of proteins for the gametocytes were first reduced with 0.1 M DTT final concentration at 60°C for 1h. MS sample preparation was performed using a FASP method (filter aided sample preparation) according to Lipecka *et al* [76]. We set aside around 10 μg of the digested proteins for the analysis of total proteomes, while the remaining samples were used for phosphopeptide enrichments. The expression of PbGSK3 was examined by western blot on purified gametocyte extracts and probed with anti-PfGSK3 (1:1000 dilution, Covalab, pab0250) and anti-Actin1 (1:2000 dilution) followed by HRP-labeled anti-rabbit (1:20000 dilution) and HRP-labeled anti-mouse (1:20000 dilution) respectively. Relative quantification of PbGSK3 in parental and Δgexp15 gametocytes was normalized using PbActin-1.

### Phosphopeptide enrichment by titanium dioxide (TiO_2_)

Phosphopeptide enrichments were carried out using Titansphere TiO_2_ Spin tips (3 mg/200 μL, Titansphere PHOS-TiO, GL Sciences Inc) on the digested proteins for each biological replicate. Briefly, the TiO_2_ Spin tips were conditioned with 20 μL of solution A (80% acetonitrile, 0,4% TFA), centrifuged at 3,000 g for 2 min and equilibrated with 20 μL of solution B (75% acetonitrile, 0,3% TFA, 25% lactic acid) followed by centrifugation at 3,000 g for 2 min. Peptides were dissolved in 20 μL of solution A, mixed with 100 μL of solution B and centrifuged at 1,000 g for 10 min. Sample was applied back to the TiO_2_ Spin tips twice more in order to increase the adsorption of the phosphopeptides to the TiO_2_. Spin tips were washed sequentially with 20μL of solution B and twice with 20μL of solution A. Phosphopeptides were eluted by the sequential addition of 50 μL of 5% NH_4_OH and 50 μL of 5% pyrrolidine. Centrifugation was carried out at 1,000 g for 5 min. Phosphopeptides were purified using GC Spin tips (GL-Tip, Titansphere, GL Sciences Inc). Briefly, the GC Spin tips were conditioned according to manufacturer’s instructions, then eluted phosphopeptides from the TiO_2_ Spin tips were added to the GC Spin tips and centrifuged at 1,000 g for 5 min. GC Spin tips were washed with 20 μL of 0.1% TFA in HPLC-grade water. Phosphopeptides were eluted with 70 μL of 80% acetonitrile, 0.1% TFA (1,000 g for 5 min) and vacuum dried.

### nanoLC-MS/MS protein identification and quantification

Peptides for the analysis of total proteomes were resuspended in 0.1% TFA in HPLC-grade water, 10% acetonitrile and 500 ng of each sample was injected in a nanoRSLC-Q Exactive PLUS (RSLC Ultimate 3000, Thermo Scientific). Phosphopeptides were resuspended in 42 μL of 0.1% TFA in HPLC-grade water and 5 μL of each sample was injected into the mass spectrometer. Samples were loaded onto a μ-precolumn (Acclaim PepMap 100 C18, cartridge, 300 μm i.d.×5 mm, 5 μm, Thermo Scientific), and were separated on a 50 cm reversed-phase liquid chromatographic column (0.075 mm ID, Acclaim PepMap 100, C18, 2 μm, Thermo Scientific). Chromatography solvents were (A) 0.1% formic acid in water, and (B) 80% acetonitrile, 0.08% formic acid. Samples were eluted from the column with the following gradient: 5% to 40% B (120 min), 40% to 80% (6 min). At 127 min, the gradient returned to 5% to re-equilibrate the column for 20 min before the next injection. One blank was run between biological replicates to prevent sample carryover. Samples eluting from the column were analyzed by data dependent MS/MS, using the top-10 acquisition method. Peptides and phosphopeptides were fragmented using higher-energy collisional dissociation (HCD). Briefly, the instrument settings were as follows: resolution was set to 70,000 for MS scans and 17,500 for the data dependent MS/MS scans in order to increase speed. The MS AGC target was set to 3.10^6^ counts with maximum injection time set to 200 ms, while MS/MS AGC target was set to 1.10^5^ with maximum injection time set to 120 ms. The MS scan range was from 400 to 2000 m/z. Dynamic exclusion was set to 30 sec duration.

The MS files were processed with the MaxQuant software version 1.5.8.3 and searched with Andromeda search engine against the database of *Mus musculus* from swissprot 07/2017 and *Plasmodium berghei* ANKA from PlasmoDB (v37) [20]. To search parent mass and fragment ions, we set a mass deviation of 4.5 ppm and 20 ppm respectively. Strict specificity for trypsin/P cleavage was required, allowing up to two missed cleavage sites. Carbamidomethylation (Cys) was set as a fixed modification, whereas oxidation (Met) and N-term acetylation were set as variable modifications. For the analysis of MS files issuing from of TiO2 enrichment, the variable modification of phosphorylation on S, T and Y were also added. The false discovery rates (FDRs) at the protein and peptide level were set to 1%. Scores were calculated in MaxQuant as described previously [77]. Match between runs was allowed. The reverse hits were removed from MaxQuant output. Proteins were quantified according to the MaxQuant label-free algorithm [77,78] using LFQ intensities and phosphopeptides according to intensity. Protein quantification was obtained using at least 2 peptides per protein.

Statistical and bioinformatic analysis, including volcano plots and clustering, were performed with Perseus software (version 1.6.0.7) freely available at Perseus [79]. For statistical comparison we set two groups: *P*. *berghei* parental and Δgexp15. Each group contained four and three biological replicates for schizonts and gametocytes respectively and each sample was run in technical triplicates.

For the total proteomes, we analyzed the proteingroups.txt file and the Phospho(STY).txt fil for the phosphoproteomes. Protein LFQ and phosphopeptides intensities were transformed in log_2_ and the site table was expanded to analyze all phosphosites separately. Proteins derived from mouse were filtered out from the analysis and the *P*. *berghei* protein and phosphosite distributions were normalized using width adjustment. We further filtered the data to keep only proteins with at least 4 valid values in the parental and/or the Δgexp15 schizonts and 3 valid values for gametocytes. Data were imputed to fill missing data points by creating a Gaussian distribution of random numbers with a standard deviation of 33% relative to the standard deviation of the measured values and 2.5 standard deviation downshift of the mean to simulate the distribution of low signal values. We performed a t-test, FDR<0.05 (250 randomizations), S0 = 0.1 for the proteomes and FDR<0.01 (250 randomizations), S0 = 0.1 for the phosphoproteomes. Hierarchical clustering of proteins and phosphosites that survived the tests was performed with Heatmapper [80] on logarithmized LFQ intensities after z-score normalization of the data, using Pearson distances.

The fold enrichments were based on GO annotations and their significance has been calculated by using hypergeometric probability test provided by Graeber lab: http://systems.crump.ucla.edu/hypergeometric/index.php

### Statistical analysis

An unpaired two-tailed non-parametric Mann–Whitney *U* test was used for the pNPP tests, comparison of the number of merozoites per schizont, gametocytes, exflagellation centers, oocysts and sporozoites. Parasitemia and survival curves were analyzed using a Wilcoxon and a log-rank (Mantel-Cox) test respectively. For growth rate, a two-way ANOVA followed by Tukey post hoc test was performed. Functional enrichments were analyzed by hypergeometric test. The criterion for a significant difference: * for p< 0.05; ** for p< 0.01, *** for p<0.001 and **** for p<0.0001. Our statistical analyses were detailed in the figure legends of each experiment. Statistical analyses were performed in GraphPad Prism 6. For the statistical analysis of the proteomes and phosphoproteomes, the details are mentioned above.

## Supporting information

S1 Fig Alignment of GEXP15 and schemes of the different regions and motifs studied. (A) Alignment between the amino acid sequences of PfGEXP15 and PbGEXP15 (BioEdit and ClustalW). (B) Schema of PfGEXP15 and PbGEXP15. The positions of the two putative RVxF motifs are shown on GEXP15 from both species and their positions are conserved. The dark grey region depicted in PfGEXP15 (8–182) corresponds to the fragment identified by the yeast two-hybrid screening of PfPP1c, and the homologous region is delineated in PbGEXP15 (4–178). The second region of PbGEXP15 (446–596, in light grey) was used in yeast two-hybrid system.
(TIF)

S2 Fig Interaction of GEXP15 with PP1 in yeast. Yeast diploids were checked on SD-LW plates (panels a) and interactions were identified by growth of undiluted and diluted (1:25 and 1:50) cultures on SD-LWH (panels b) and SD-LWHA (panels c). (A) pGADT7-PbGEXP15 _4–590_ was mated with pGBKT7-PfPP1c (lane 1), with pGBKT7-Laminin (lane 2) and with pGBKT7 (lane 3). The mating of yeasts transformed with pGADT7 and pGBKT7-PfPP1c was used as a negative control (lane 4). (B) pGADT7-PbGEXP15 _4–178_ was mated in the same manner as above (lanes 1, 2 and 3). Lanes 4 and 5 represent the mating of pGBKT7-PfPP1c with pGADT7-PbGEXP15 _446–596_ and pGADT7-PbGEXP15 _4–178 KKKKKAQA_ respectively. (C) pGADT7-PbGEXP15 _4–590_ (lane 1), pGADT7-PbGEXP15 _4–178_ (lane 2), pGADT7-PbGEXP15 _446–596_ (lane 3) and pGADT7-PbGEXP15 _4–178 KKKKKAQA_ (lane 4) were mated with pGBKT7-PfPP1c F255A F256A (annotated PfPP1c FF). The mating of yeasts transformed with pGADT7 and PGBKT7-PfPP1c F255A F256A was used as a control (lane 5). (D) Diagnostic RT-PCR of the different exogenous PfPP1c and PbGEXP15 in yeast. The amplification of the intronic gene *tub1*, on yeast genomic DNA, was used as control (lane 1). cDNAs were obtained after reverse transcription of total RNA from yeasts transfected with pGBKT7-PfPP1c (lanes 2, 3), PGBKT7-PfPP1c F255A F256A (lanes 4, 5), pGADT7-PbGEXP15 _4–590_ (lanes 6, 7), pGADT7-PbGEXP15 _4–178_ (lanes 8, 9), pGADT7-PbGEXP15 _4–178 KKKKKAQA_ (lanes 10, 11) and pGADT7-PbGEXP15 _446–596_ (lanes 12, 13). RT-PCRs were performed using primers p13-p14 for TUB1 (lanes 1, 2, 4, 6, 8, 10, 12), p11-p12 for PfPP1c and PfPP1c F255A F256A (lanes 3, 5), p8-p9 for PbGEXP15 _4–590_, PbGEXP15 _4–178_ and PbGEXP15 _4–178 KKKKKAQA_ (lanes 7, 9, 11) and p8-p10 for PbGEXP15 _446–596_ (lane 13). Schematic representations indicate positions of the different primers.
(TIF)

S3 Fig Strategy and genotyping of PbPP1c-mCherry and PbGEXP15-mCherry. Schematic representations of the mCherry tag integration strategy by single homologous recombination into endogenous *pbpp1c* (A) or *pbgexp15* (B) locus and genotype analyses. The selectable marker (TgDHFR), the mCherry tag, the PCR primers and the positions of the restriction site (white dots) used to linearize the constructs are indicated. PCRs were performed on parental and transfected genomic DNA using the indicated primer combinations. Immunoblot detection of PbPP1-mCherry (C) and PbGEXP15-mCherry (D) in parental (lanes 1) and transfected *P*. *berghei* parasites (lanes 2), probed with anti-mCherry. (E) Immunoblot detection of wild PbGEXP15 in *P*. *berghei* parasites with anti-GEXP15 antisera (lane 1) and pre-immune sera (lane 2). Of note, heavy-chain antibodies were detected in both conditions.
(TIF)

S4 Fig Strategy and analyses of the PbGEXP15 knock-out. (A) Schematic representation of the knock-out strategy by double homologous recombination of the endogenous *pbgexp15* locus and genotyping PCRs. The selectable marker (TgDHFR) and the PCR primers are indicated. The presence of the endogenous locus of PbGEXP15 was demonstrated by PCR using primers p15-p17 on parental, Δgexp15cl1 and Δgexp15cl2 genomic DNA (lanes 1, 4, 7 respectively). Integrations at the 5’ and 3’ ends were verified for parental (lanes 2, 3), Δgexp15cl1 (lanes 5, 6) and Δgexp15cl2 (lanes 8, 9) with primers p30-p31 and p32-p33 respectively. (B) Western blot analysis of PbGEXP15 expression in parental (lanes 1 and 4), Δgexp15cl1 (lanes 2 and 5) and Δgexp15cl2 parasites (lanes 3 and 6). Immunoblot (IB) was probed with anti-GEXP15 antisera (lanes 1, 2 and 3) or pre-immune sera (lanes 4, 5 and 6). Anti-Actin1 was used as a loading control. (C) Representative photographs of infected C57BL/6 mice brains. Analysis of the breakdown of blood brain barrier of mouse infected by parental or Δgexp15cl1 parasites was carried out using Evans blue (day 6 p.i).
(TIF)

S5 Fig Supplemental analyses of proteomes and phosphosproteomes in parental and Δgexp15 schizont and gametocyte stages. (A) Venn diagram showing the overlaps between the IP/MS of PbPP1, PfPP1 interactome, and the proteome and phosphoproteome in Δgexp15 schizonts. (B) Representative western blot analysis of PbGSK3 expression in parental (lanes 1) and Δgexp15 gametocytes (lanes 2). Immunoblot was probed with anti-GSK3 (left panel) and anti-Actin1 (right panel). (C) Relative expression of PbGSK3 to PbActin-1 was normalized in parental gametocytes. Data are presented as mean ± SD of two independent experiments.
(TIF)

S1 Table Identified proteins after PbGEXP15-mCherry immunoprecipitation in *P*. *berghei* schizonts. The table shows the protein name, accession number and molecular weight of identified proteins and the number of peptides and spectra identified for each experiment are indicated. Three experiments were performed with mCherry antibodies on WT schizonts as negative control and PbGEXP15-mCherry schizonts. Here, we present the protein co-immunoprecipitated with PbGEXP15-mCherry in at least two experiments with peptides ≥ 2 and with peptides and spectra ≥ 2 fold compared with the control strain. Blank cells represent 0 peptide/spectrum identified. Percentage of sequence coverage, peptide sequence, number of shared peptides and other proteins are indicated in reduced columns. In the second sheet, all proteins immunoprecipitated are shown.
(XLSX)

S2 Table Identified proteins after PbGEXP15-mCherry immunoprecipitation in *P*. *berghei* gametocytes. The table shows the protein name, accession number and molecular weight of identified proteins and the number of peptides and spectra identified for each experiment are indicated. Two experiments were performed with mCherry antibodies on WT gametocytes as negative control and PbGEXP15-mCherry gametocytes, Here, we present the protein co-immunoprecipitated with PbGEXP15-mCherry in two experiments with peptides ≥ 2 and with peptides and spectra ≥ 2 fold compared with the control strain. Blank cells represent 0 peptide/spectrum identified. Percentage of sequence coverage, peptide sequence, number of shared peptides and other proteins are indicated in reduced columns. In the second sheet, all proteins immunoprecipitated are shown.
(XLS)

S3 Table Identified proteins after PbPP1-HA immunoprecipitation in *P*. *berghei* schizonts. The table shows the protein name, accession number and molecular weight of identified proteins and the number of peptides and spectra identified for each experiment are indicated. Three experiments were performed with HA antibodies on WT schizonts as negative control and PbPP1-HA schizonts. Here, we present the protein co-immunoprecipitated with PbPP1-HA in at least two experiments with peptides ≥ 2 and with peptides and spectra ≥ 2 fold compared with the control strain. Blank cells represent 0 peptide/spectrum identified. Percentage of sequence coverage, peptide sequence, number of shared peptides and other proteins are indicated in reduced columns. In the second sheet, all proteins immunoprecipitated are shown.
(XLSX)

S4 Table Table of proteins of *P*. *berghei* parental and Δgexp15 schizonts. This table reports the proteins quantified in parental and Δgexp15 schizonts. The values reported under parental 1–4 and Δgexp15 1–4 correspond to logaritmic labelfree intensity (LFQ), "MS/MS counts" is the total number of assigned fragmented spectra, "peptides" is the number of peptides associated with each protein group. Student’s T-test significant indicates the proteins that survived the test after multiple testing using FDR<0.05. The fold change is indicated and a color gradient from green to red represents the proteins under and over-abundant respectively in Δgexp15 schizonts. We report the p and q value of the T test between parental and Δgexp15, as well as the differences of the intensities. We report biological process associated to each protein when known based on manual and COG annotation. For proteins with a significant differential abundance in parental and Δgexp15 schizonts (FDR<0.05), *P*. *falciparum* orthologs are added when known (/ indicates no ortholog). ^a^ Proteins described to be partners of the PP1 network in *P*. *falciparum* [17] and in *P*. *berghei* (this study) are indicated by X.
(XLSX)

S5 Table Phosphosites identified in the *P*. *berghei* parental and Δgexp15 schizonts. This table reports the phosphosites quantified in parental and Δgexp15 schizonts. For each phosphosite we report the normalised log2 of the intensity values. The T test was performed between the parental and Δgexp15 schizonts. P-value, q value and difference of the average of the intensities are reported. Student’s T-test significant indicates the 166 phosphosites that survived the test after multiple testing using FDR<0.01. The fold change is indicated and a color gradient from green to red represents the phosphopeptides hypo- and hyperphosphorylated respectively in Δgexp15 schizonts. Amino Acid indicates if the phosphorylation occurs on S, T or Y, the position indicates the amino acid position in the protein. Multiplicity is the number of phosphorylation identified simultaneously on that peptide. Localization probability is the probability of correct phosphosite attribution (1 indicates 100%). Positions within the proteins indicate all the phosphorylation sites identified on the protein. Sequence window contains the sequence of the 15 amino acid before and after in the phosphosite. We report biological process associated to each protein when known based on manual and COG annotation. For the phosphosites with a significant differential in parental and Δgexp15 schizonts (FDR<0.01), *P*. *falciparum* orthologs are added when known (/ indicates no ortholog). ^a^ Proteins previously described to be partners or potential member of the PP1 network in *P*. *falciparum* [17] and in *P*. *berghei* (this study) are indicated by X. ^b^ Phosphoproteins identified in published *P*. *berghei* phosphoproteome studies [81,82] are reported (X: previously identified; / indicates no ortholog). ^c^ Phosphoproteins identified in published *P*. *falciparum* phosphoproteome studies [83–85] are reported (X: previously identified; / indicates no ortholog).
(XLSX)

S6 Table Table of proteins of *P*. *berghei* parental and Δgexp15 gametocytes. This table reports the proteins quantified in parental and Δgexp15 gametocytes. The values reported under parental 1–3 and Δgexp15 1–3 correspond to logaritmic labelfree intensity (LFQ), "MS/MS counts" is the total number of assigned fragmented spectra, "peptides" is the number of peptides associated with each protein group. Student’s T-test significant indicates the proteins that survived the test after multiple testing using FDR<0.05. The fold change is indicated and a color gradient from green to red represents the proteins under and over-abundant respectively in Δgexp15 gametocytes. We report the p and q value of the T test between parental and Δgexp15, as well as the differences of the intensities. We report biological process associated to each protein when known based on manual and COG annotation. For proteins with a significant differential abundance in parental and Δgexp15 gametocytes (FDR<0.05), *P*. *falciparum* orthologs are added when known. ^a^ Sex is indicated when the proteins are enriched in male and female gametocyte transcriptomes or proteomes in *P*. *berghei* [41] and *P*. *falciparum* [40,42] studies.
(XLSX)

S7 Table Phosphosites identified in the *P*. *berghei* parental and Δgexp15 gametocytes. This table reports the phosphosites quantified in parental and Δgexp15 gametocytes. For each phosphosite we report the normalised log2 of the intensity values. The T test was performed between the parental and Δgexp15 gametocytes. P-value, q value and difference of the average of the intensities are reported. Student’s T-test significant indicates the 9 phosphosites that survived the test after multiple testing using FDR<0.01. The fold change is indicated and a color gradient from green to red represents the phosphopeptides hypo- and hyperphosphorylated respectively in Δgexp15 gametocytes. Amino Acid indicates if the phosphorylation occurs on S, T or Y, the position indicates the amino acid position in the protein. Multiplicity is the number of phosphorylation identified simultaneously on that peptide. Localization probability is the probability of correct phosphosite attribution (1 indicates 100%). Positions within the proteins indicate all the phosphorylation sites identified on the protein. Sequence window contains the sequence of the 15 amino acid before and after in the phosphosite. We report biological process associated to each protein when known based on manual and COG annotation. For the phosphosites with a significant differential in parental and Δgexp15 gametocytes (FDR<0.01), *P*. *falciparum* orthologs are added when known (/ indicates no ortholog). ^a^ Phosphoproteins identified in published *P*. *berghei* phosphoproteome studies [81,82] are reported (X: previously identified; / indicates no ortholog). ^b^ Phosphoproteins identified in published *P*. *falciparum* phosphoproteome studies [83–85] are reported (X: previously identified; / indicates no ortholog). ^c^ Sex is indicated when the proteins are enriched in male and female gametocyte transcriptomes or proteomes in *P*. *berghei* [41] and *P*. *falciparum* [40,42] studies.
(XLSX)

S8 Table List of primers used in this study. (XLSX)
