# Supplementary figures and images for: Essential role of GEXP15, a specific Protein Phosphatase type 1 partner, in Plasmodium berghei in asexual erythrocytic proliferation and transmission
Source: PLoS Pathog. 2019 Jul 26;15(7):e1007973. doi: 10.1371/journal.ppat.1007973 (PMC6685639; doi:10.1371/journal.ppat.1007973)

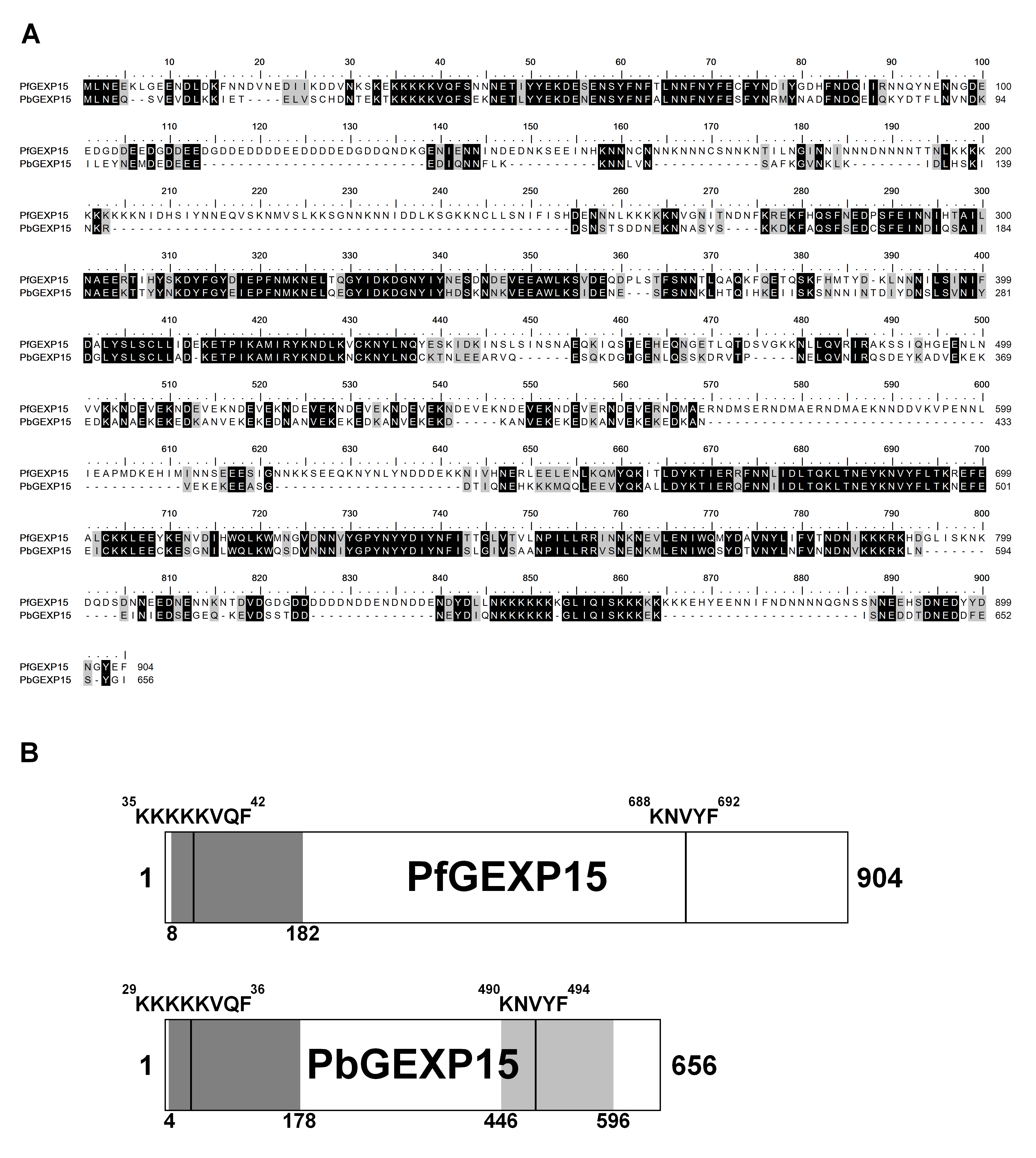

Supplement: S1 Fig — (A) Alignment between the amino acid sequences of PfGEXP15 and PbGEXP15 (BioEdit and ClustalW). (B) Schema of PfGEXP15 and PbGEXP15. The positions of the two putative RVxF motifs are shown on GEXP15 from both species and their positions are conserved. The dark grey region depicted in PfGEXP15 (8–182) corresponds to the fragment identified by the yeast two-hybrid screening of PfPP1c, and the homologous region is delineated in PbGEXP15 (4–178). The second region of PbGEXP15 (446–596, in light grey) was used in yeast two-hybrid system. (TIF) [file ppat.1007973.s001.tif]

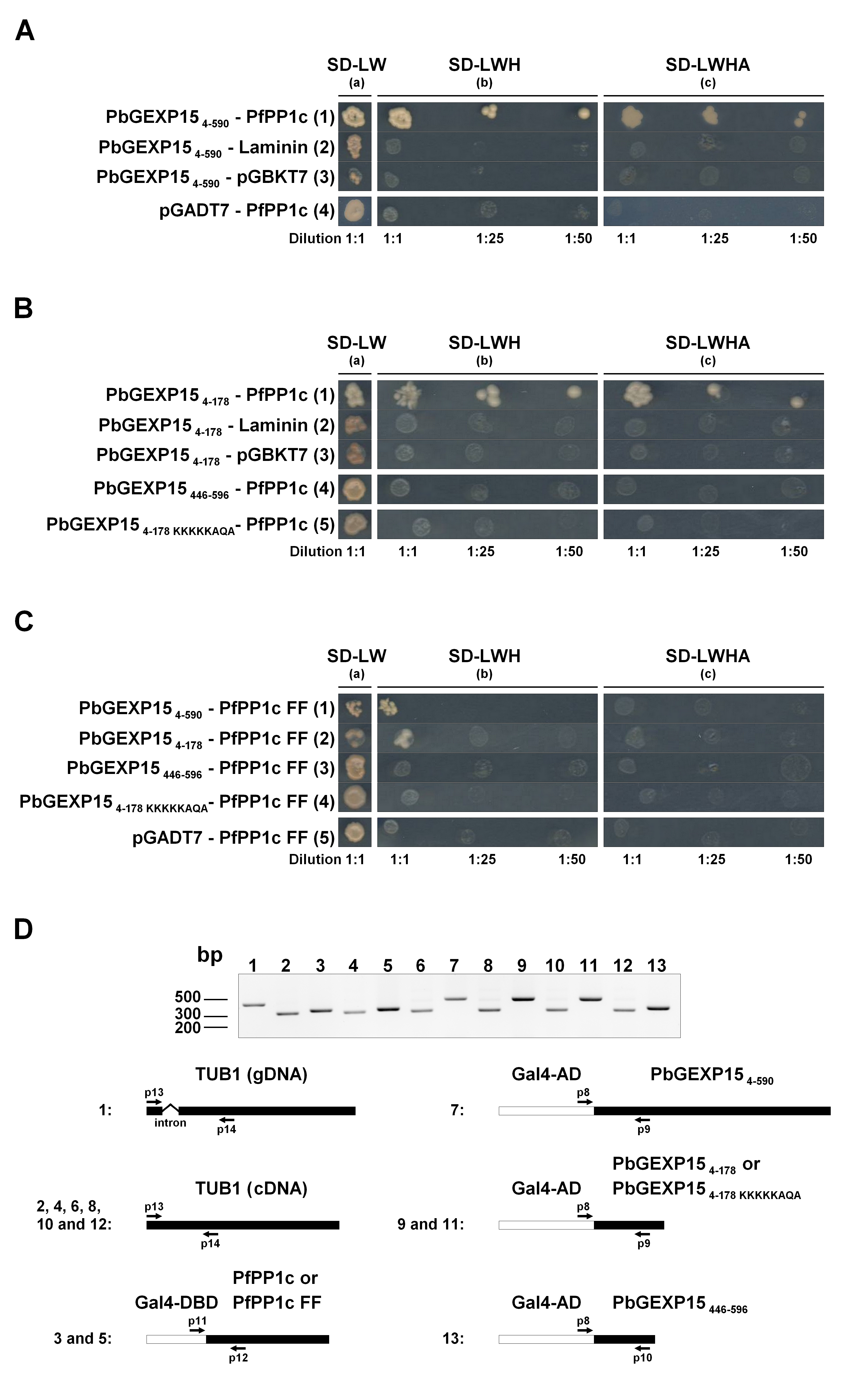

Supplement: S2 Fig — Yeast diploids were checked on SD-LW plates (panels a) and interactions were identified by growth of undiluted and diluted (1:25 and 1:50) cultures on SD-LWH (panels b) and SD-LWHA (panels c). (A) pGADT7-PbGEXP15 4–590 was mated with pGBKT7-PfPP1c (lane 1), with pGBKT7-Laminin (lane 2) and with pGBKT7 (lane 3). The mating of yeasts transformed with pGADT7 and pGBKT7-PfPP1c was used as a negative control (lane 4). (B) pGADT7-PbGEXP15 4–178 was mated in the same manner as above (lanes 1, 2 and 3). Lanes 4 and 5 represent the mating of pGBKT7-PfPP1c with pGADT7-PbGEXP15 446–596 and pGADT7-PbGEXP15 4–178 KKKKKAQA respectively. (C) pGADT7-PbGEXP15 4–590 (lane 1), pGADT7-PbGEXP15 4–178 (lane 2), pGADT7-PbGEXP15 446–596 (lane 3) and pGADT7-PbGEXP15 4–178 KKKKKAQA (lane 4) were mated with pGBKT7-PfPP1c F255A F256A (annotated PfPP1c FF). The mating of yeasts transformed with pGADT7 and PGBKT7-PfPP1c F255A F256A was used as a control (lane 5). (D) Diagnostic RT-PCR of the different exogenous PfPP1c and PbGEXP15 in yeast. The amplification of the intronic gene tub1, on yeast genomic DNA, was used as control (lane 1). cDNAs were obtained after reverse transcription of total RNA from yeasts transfected with pGBKT7-PfPP1c (lanes 2, 3), PGBKT7-PfPP1c F255A F256A (lanes 4, 5), pGADT7-PbGEXP15 4–590 (lanes 6, 7), pGADT7-PbGEXP15 4–178 (lanes 8, 9), pGADT7-PbGEXP15 4–178 KKKKKAQA (lanes 10, 11) and pGADT7-PbGEXP15 446–596 (lanes 12, 13). RT-PCRs were performed using primers p13-p14 for TUB1 (lanes 1, 2, 4, 6, 8, 10, 12), p11-p12 for PfPP1c and PfPP1c F255A F256A (lanes 3, 5), p8-p9 for PbGEXP15 4–590, PbGEXP15 4–178 and PbGEXP15 4–178 KKKKKAQA (lanes 7, 9, 11) and p8-p10 for PbGEXP15 446–596 (lane 13). Schematic representations indicate positions of the different primers. (TIF) [file ppat.1007973.s002.tif]

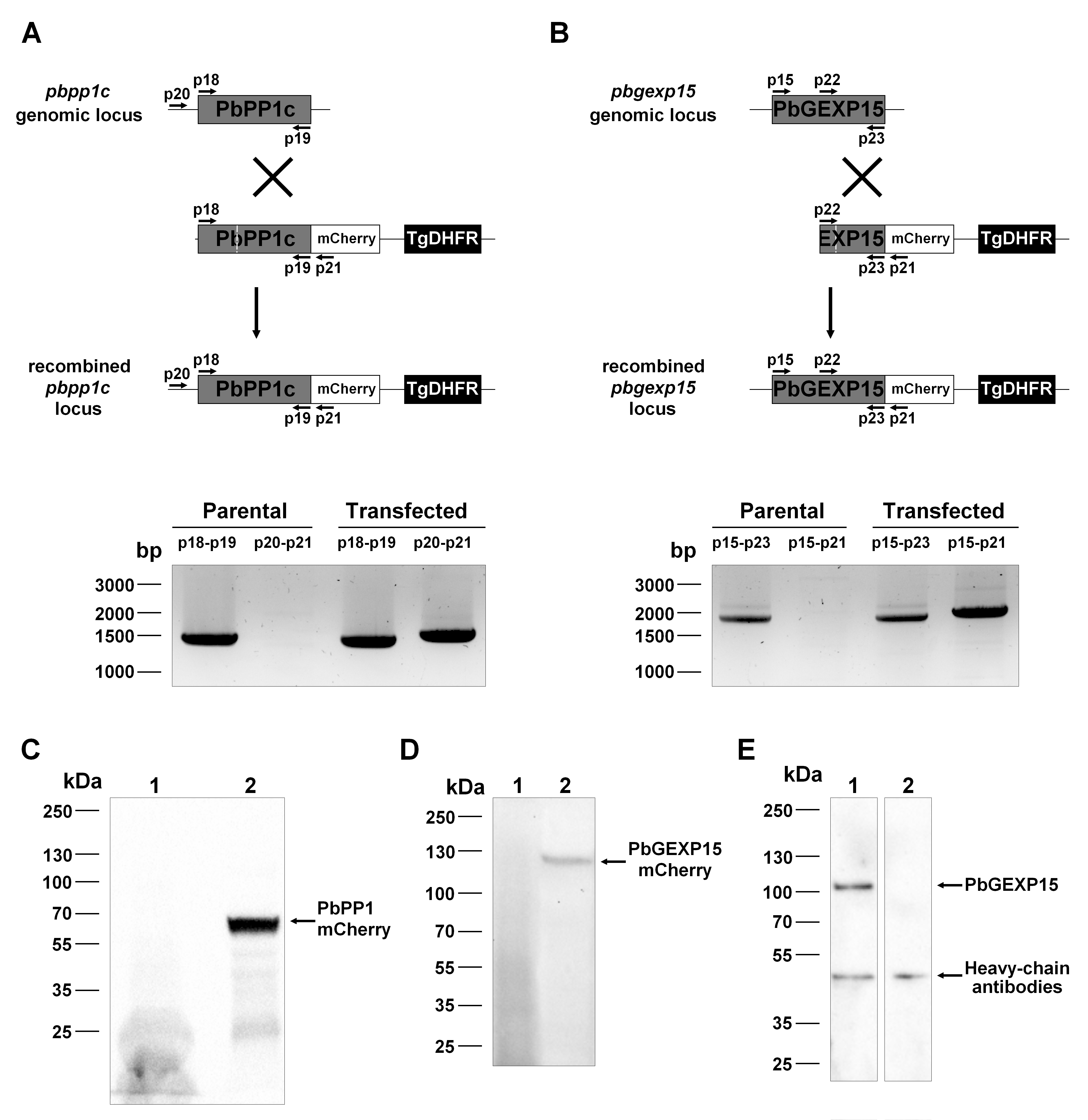

Supplement: S3 Fig — Schematic representations of the mCherry tag integration strategy by single homologous recombination into endogenous pbpp1c (A) or pbgexp15 (B) locus and genotype analyses. The selectable marker (TgDHFR), the mCherry tag, the PCR primers and the positions of the restriction site (white dots) used to linearize the constructs are indicated. PCRs were performed on parental and transfected genomic DNA using the indicated primer combinations. Immunoblot detection of PbPP1-mCherry (C) and PbGEXP15-mCherry (D) in parental (lanes 1) and transfected P. berghei parasites (lanes 2), probed with anti-mCherry. (E) Immunoblot detection of wild PbGEXP15 in P. berghei parasites with anti-GEXP15 antisera (lane 1) and pre-immune sera (lane 2). Of note, heavy-chain antibodies were detected in both conditions. (TIF) [file ppat.1007973.s003.tif]

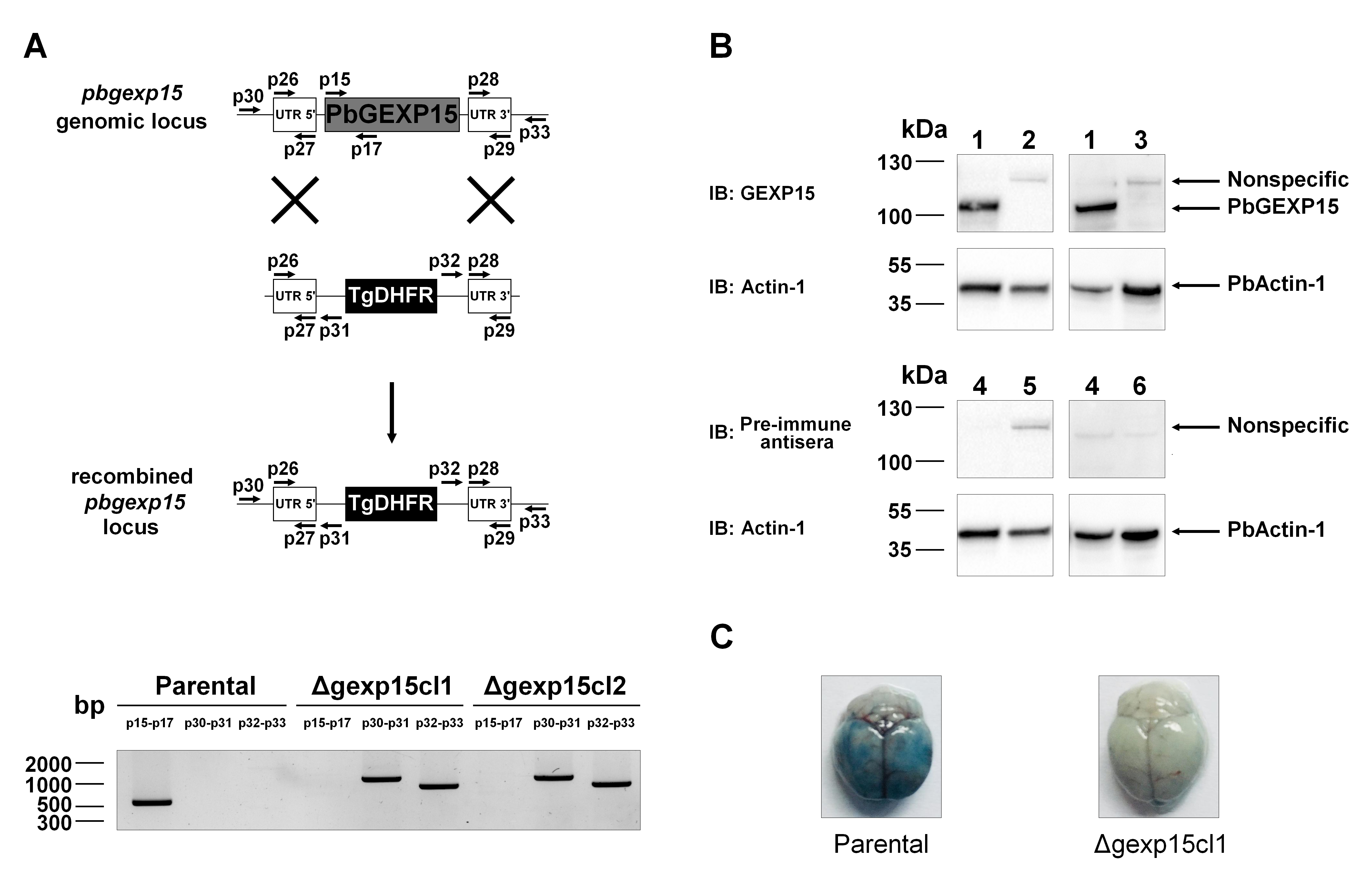

Supplement: S4 Fig — (A) Schematic representation of the knock-out strategy by double homologous recombination of the endogenous pbgexp15 locus and genotyping PCRs. The selectable marker (TgDHFR) and the PCR primers are indicated. The presence of the endogenous locus of PbGEXP15 was demonstrated by PCR using primers p15-p17 on parental, Δgexp15cl1 and Δgexp15cl2 genomic DNA (lanes 1, 4, 7 respectively). Integrations at the 5’ and 3’ ends were verified for parental (lanes 2, 3), Δgexp15cl1 (lanes 5, 6) and Δgexp15cl2 (lanes 8, 9) with primers p30-p31 and p32-p33 respectively. (B) Western blot analysis of PbGEXP15 expression in parental (lanes 1 and 4), Δgexp15cl1 (lanes 2 and 5) and Δgexp15cl2 parasites (lanes 3 and 6). Immunoblot (IB) was probed with anti-GEXP15 antisera (lanes 1, 2 and 3) or pre-immune sera (lanes 4, 5 and 6). Anti-Actin1 was used as a loading control. (C) Representative photographs of infected C57BL/6 mice brains. Analysis of the breakdown of blood brain barrier of mouse infected by parental or Δgexp15cl1 parasites was carried out using Evans blue (day 6 p.i). (TIF) [file ppat.1007973.s004.tif]

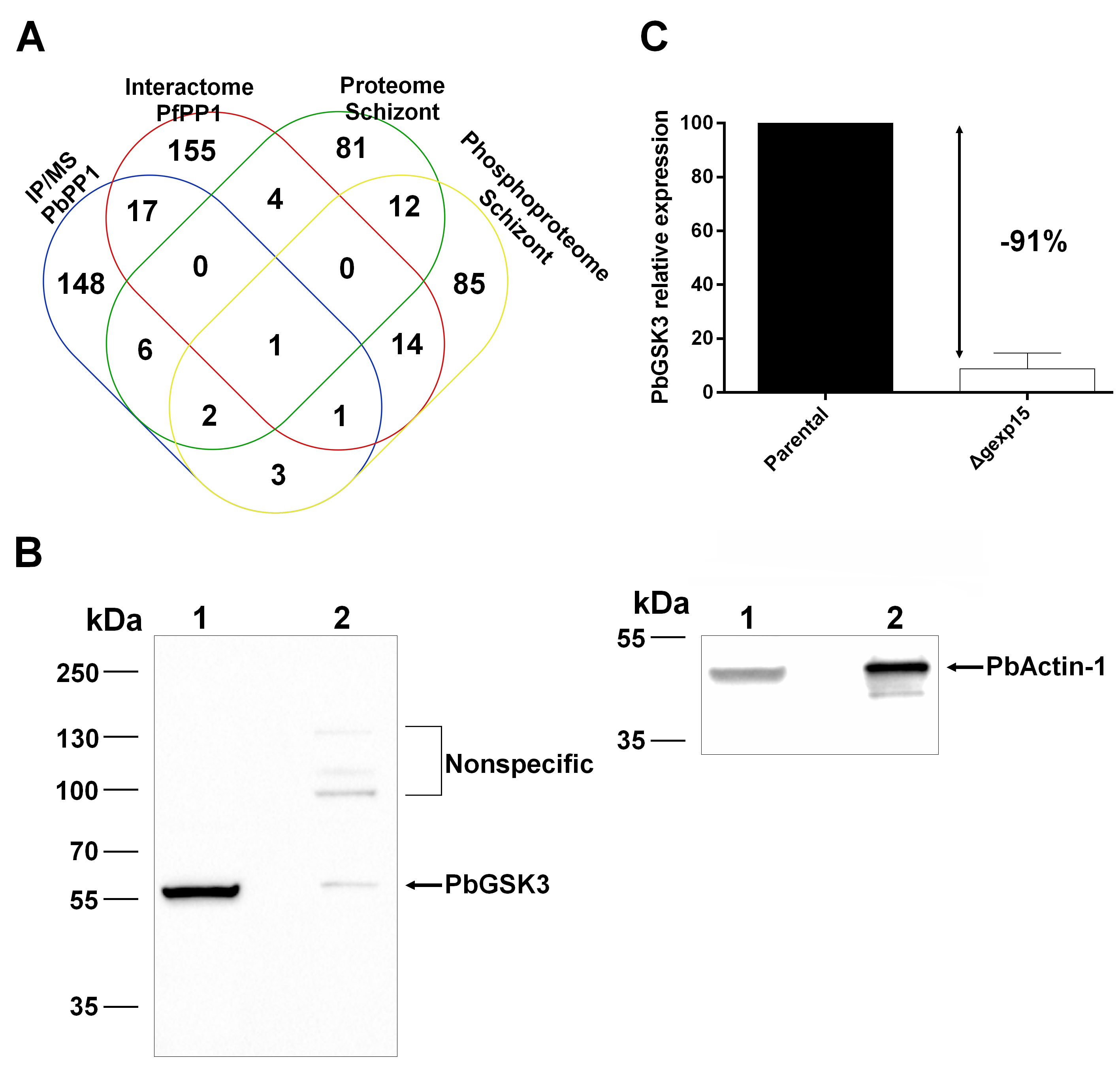

Supplement: S5 Fig — (A) Venn diagram showing the overlaps between the IP/MS of PbPP1, PfPP1 interactome, and the proteome and phosphoproteome in Δgexp15 schizonts. (B) Representative western blot analysis of PbGSK3 expression in parental (lanes 1) and Δgexp15 gametocytes (lanes 2). Immunoblot was probed with anti-GSK3 (left panel) and anti-Actin1 (right panel). (C) Relative expression of PbGSK3 to PbActin-1 was normalized in parental gametocytes. Data are presented as mean ± SD of two independent experiments. (TIF) [file ppat.1007973.s005.tif]
